# Gut-brain communication by distinct sensory neurons differently controls feeding and glucose metabolism

**DOI:** 10.1016/j.cmet.2021.05.002

**Published:** 2021-07-06

**Authors:** Diba Borgmann, Elisa Ciglieri, Nasim Biglari, Claus Brandt, Anna Lena Cremer, Heiko Backes, Marc Tittgemeyer, F. Thomas Wunderlich, Jens C. Brüning, Henning Fenselau

**Affiliations:** 1 Synaptic Transmission in Energy Homeostasis Group, Max Planck Institute for Metabolism Research, Gleueler Strasse 50, 50931 Cologne, Germany; 2 Translational Neurocircuitry Group, Max Planck Institute for Metabolism Research, Gleueler Strasse 50, 50931 Cologne, Germany; 3 Center for Anatomy II, Neuroanatomy, University Hospital Cologne, Joseph-Stelzmann Str. 9, 50937 Cologne, Germany; 4 Center for Endocrinology, Diabetes and Preventive Medicine (CEDP), University Hospital Cologne, Kerpener Strasse 26, 50937 Cologne, Germany; 5 Max Planck Institute for Metabolism Research, Department of Neuronal Control of Metabolism, Gleueler Strasse 50, 50931 Cologne, Germany; 6 Excellence Cluster on Cellular Stress Responses in Aging Associated Diseases (CECAD), University of Cologne, Joseph-Stelzmann-Straße 26, Cologne 50931, Germany; 7 Center of Molecular Medicine Cologne (CMMC), University of Cologne, Robert-Koch-Straße 21, 50931 Cologne, Germany

**Keywords:** gut-brain axis, sensory neurons, vagus nerve, glucose metabolism, chemogenetics, Dre-recombinase, intersectional genetics, nodose ganglion, dorsal root ganglion, appetite

## Abstract

Sensory neurons relay gut-derived signals to the brain, yet the molecular and functional organization of distinct populations remains unclear. Here, we employed intersectional genetic manipulations to probe the feeding and glucoregulatory function of distinct sensory neurons. We reconstruct the gut innervation patterns of numerous molecularly defined vagal and spinal afferents and identify their downstream brain targets. Bidirectional chemogenetic manipulations, coupled with behavioral and circuit mapping analysis, demonstrated that gut-innervating, glucagon-like peptide 1 receptor (GLP1R)-expressing vagal afferents relay anorexigenic signals to parabrachial nucleus neurons that control meal termination. Moreover, GLP1R vagal afferent activation improves glucose tolerance, and their inhibition elevates blood glucose levels independent of food intake. In contrast, gut-innervating, GPR65-expressing vagal afferent stimulation increases hepatic glucose production and activates parabrachial neurons that control normoglycemia, but they are dispensable for feeding regulation. Thus, distinct gut-innervating sensory neurons differentially control feeding and glucoregulatory neurocircuits and may provide specific targets for metabolic control.

## Highlights

• Intersectional mapping of sensory neurons identifies distinct gut innervation patterns
• Gut-innervating GLP1R+ vagal afferents relay anorexigenic signals to brainstem neurons
• Gut-innervating GPR65+ vagal afferent stimulation increases hepatic glucose production
• GLP1R+ vagal afferent activity is required to control glycemia during feeding

## Graphical abstract

**Figure:**
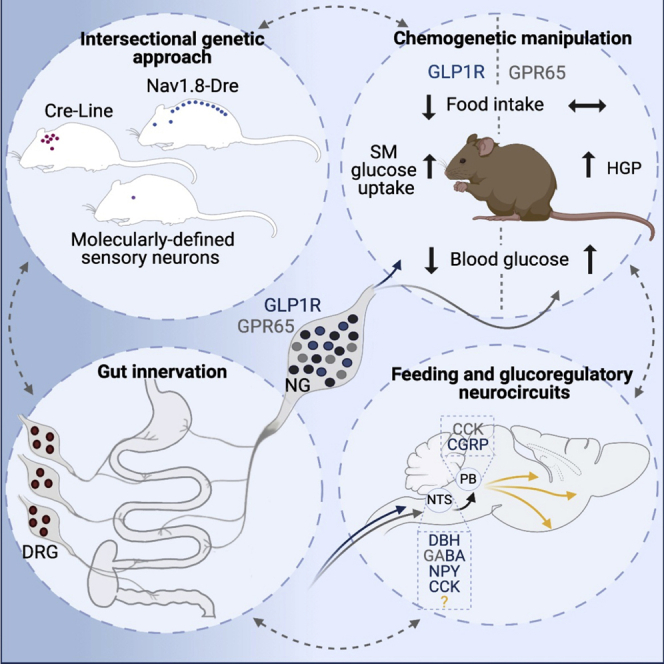

## Teaser

Borgmann et al. devise an intersectional genetic approach to probe the contribution of molecularly defined sensory neurons in gut-brain communication. They identify that distinct gut-innervating vagal afferents differentially control food intake and peripheral glucose metabolism, and engage distinct downstream circuits in the brain.

## Introduction

Gut-innervating sensory neurons are a major afferent pathway of the gut-brain axis (Clemmensen et al., 2017; Kim et al., 2018; Soty et al., 2017). Conventionally, the function of these neurons is to transmit nutrient-related signals from the gut to the brain upon food consumption to induce, in turn, satiation and adaptive glucoregulatory responses so that meal termination and blood glucose levels are controlled (Kim et al., 2018; Schwartz et al., 2000). Consistent with this, nutrient administration directly into the stomach or duodenum reduces food intake and adapts insulin sensitivity, and these regulatory actions are prevented by ablating sensory neurons (Liebling et al., 1975; Reidelberger et al., 1983; Wang et al., 2008; Welch et al., 1988; Yox and Ritter, 1988). Notably, impairment of this feedback communication has been associated with systemic metabolic dysfunction. Specifically, in obesity, impaired responses of sensory neurons to gut delivery of nutrients have been attributed to overeating, body weight gain, and insulin resistance (Boyd et al., 2003; Cheung et al., 2009; Covasa, 2010; Wang et al., 2008).

Despite the established importance of sensory neurons in gut-brain communication, it remains unclear which of these cells actually participate in the regulation of feeding and blood glucose levels. Nevertheless, various populations, which are residing in nodose ganglia (NG; vagal afferents) and dorsal root ganglia (DRG; spinal afferents), are likely important as suggested by numerous compelling studies. First, as determined through classical tracing and histological studies, peripheral terminals from different vagal and spinal afferents innervate the organs of the gastrointestinal (GI) tract (Berthoud et al., 1995, Berthoud et al., 2004; Berthoud and Powley, 1992; Phillips et al., 1997; Spencer et al., 2014). Importantly, the distinct tissue innervations are generally believed to reflect the function of different populations (Berthoud and Neuhuber, 2000). Second, different sensory neurons respond to gut-derived signals, such as gastric distension, nutrients, or hormones, which are released from enteroendocrine cells, including GLP-1 and cholecystokinin (CCK) (Blackshaw and Grundy, 1990; Phillips and Powley, 2000; Richards et al., 1996; Rüttimann et al., 2009; Williams et al., 2016). Third, surgical dissection of vagus nerve branches (vagotomy) innervating disparate GI tract organs alters meal termination and glucose metabolism (Berthoud and Neuhuber, 2000; Duraffourd et al., 2012; Walls et al., 1995; Wang et al., 2008). Similarly, varying metabolic effects are observed from administration of capsaicin, which compromises transient receptor potential vanilloid 1 (TRPV1)-expressing sensory neurons of vagal and spinal origin (Berthoud et al., 1997; De Vadder et al., 2014; Phillips and Powley, 2000; Ritter and Ladenheim, 1985; van de Wall et al., 2005). Fourth, numerous distinct sensory neuron populations in NG and DRG have been revealed by G protein-coupled-receptor-expression-based and single-cell RNA sequencing studies (Bai et al., 2019; Hockley et al., 2019; Kupari et al., 2019; Usoskin et al., 2015; Williams et al., 2016). RNA sequencing studies have also identified genetic markers for vagal and spinal afferents that innervate GI tract organs (Bai et al., 2019; Hockley et al., 2019; Williams et al., 2016).

Together, the above findings indicate that distinct sensory neurons, which innervate different organs/tissues of the GI tract, respond to different gut-derived signals and that their neuronal activation contributes to the regulation of feeding and glucose metabolism. In agreement with this, recent imaging studies in anesthetized animals have revealed the *in vivo* activity regulation of genetically identified sensory neurons. Specifically, calcium imaging of vagal ganglia showed that GLP1R-expressing neurons are selectively activated by stomach stretch, whereas perfusion of nutrients or high osmolar solutions into the small intestine activates GPR65-expressing neurons (Tan et al., 2020; Williams et al., 2016). Additionally, acute organ- or cell-type-specific stimulation of vagal afferents has been shown to be sufficient to alter food intake (Bai et al., 2019; Chen et al., 2020; Han et al., 2018). Opto- or chemogenetic stimulation of upper-gut-innervating, GLP1R-expressing, or oxytocin-receptor-expressing vagal afferents reduced feeding (Bai et al., 2019; Brierley et al., 2021; Han et al., 2018), whereas chemogenetically stimulating vagal afferents that synaptically engage tyrosine-hydroxylase-expressing neurons in the nucleus of the solitary tract (NTS) increased feeding (Chen et al., 2020).

However, the identity of the gut-innervating sensory neuron populations that participate in the acute regulation of glucose metabolism remains unclear. Furthermore, although gut-derived stimuli have been demonstrated to activate distinct sensory neurons, the contribution of their activation to the physiological regulation of feeding and glucoregulatory responses along with the pertaining downstream circuits in the brain remain poorly understood. A major obstacle in deciphering their functional neurocircuits has been the technical difficulties associated with cell-type-specific targeting sensory neurons in NG and DRG, which are not only small in size but also difficult to access because of their locations close to the carotid artery and vertebral column, respectively.

To overcome these issues, we have designed an intersectional (dual-recombinase) genetic approach that allows mapping and manipulating molecularly defined sensory neurons. Subsequent anatomical studies revealed the different gut innervation patterns of numerous populations and identified their central projections. Moreover, through the use of transgenic mouse lines that allow for intersectional expression of hM3Dq and hM4Di, for acute chemogenetic activation and inhibition, respectively, we employed two non-overlapping, vagal afferents that selectively innervate the gut. These studies have uncovered detailed insights about their feeding and glucoregulatory function as well as the downstream neurocircuits.

## Results

### Intersectional genetic targeting molecularly defined sensory neurons

**Figure 1:**
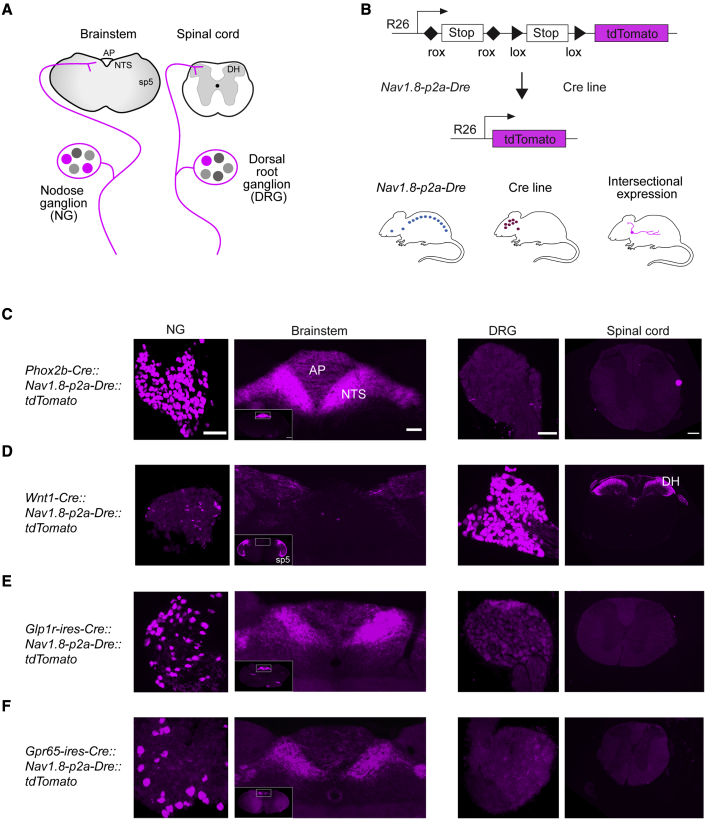
Intersectional genetic targeting of molecularly defined sensory neurons (A) Schematic of sensory neuron locations and their central projections. Nodose ganglia (NG; vagal afferents) neurons project to the brainstem, where they innervate the nucleus of the solitary tract (NTS) and the area postrema (AP). Dorsal root ganglia (DRG; spinal afferents) neurons innervate the dorsal horn (DH) of the spinal cord. (B) Breeding schematic for triple transgenic mice. Dre-/Cre-dependent tdTomato reporter mice (Madisen et al., 2015) were crossed with *Nav1.8-p2a-Dre* mice and Cre-expressing mouse lines. Dre and Cre recombinases excise rox and lox sites, respectively, allowing expression of tdTomato in discrete sensory neuron populations. (C–F) tdTomato (magenta) expression in NG, brainstem, DRG, and spinal cord in triple transgenic mice derived from *Phox2b-Cre* (C), *Wnt1-Cre* (D), *Glp1r-ires-Cre* (E), and *Gpr65-ires-Cre* (F) mice. Spinal trigeminal nucleus, sp5. Spinal dorsal horn, DH. Scale bars represent 100 μm (NG and DRG), 100 μm (brainstem; 500 μm inset), and 200 μm (spinal cord). See also Figure S1 and Table S1.

To investigate the functional neurocircuits of gut-innervating sensory neurons, we sought to develop a genetic approach that allows non-invasive targeting individual vagal and spinal afferent populations (Figures 1A and 1B). For this purpose, we employed three sets of mouse lines. The first one is the *Nav1.8-p2a-Dre* line, which expresses the Dre-recombinase under control of the promoter region of the *Scn10a* gene, which encodes Nav1.8 (Figure S1A). We reasoned that Nav1.8, a sodium channel that is exclusively expressed in sensory neurons (Akopian et al., 1996; Djouhri et al., 2003), including those innervating the gut (Bai et al., 2019; Gautron et al., 2011), would enable precise and reproducible intersectional targeting of distinct vagal and spinal afferents. To validate this newly developed line, we crossed *Nav1.8-p2a-Dre* mice with mice that express the fluorophore ZsGreen after Dre-dependent excision of a rox-flanked STOP cassette from the ubiquitous Rosa26 locus (rox: Dre-recombinase recognition site; *Rosa26-rox-STOP-rox-ZsGreen* mice) (Löhr et al., 2018). In the resulting *Nav1.8-p2a-Dre::ZsGreen* mice, we assessed NG and DRG using fluorescent *in situ* hybridization (FISH). The vast majority of Nav1.8+ (*Scn10a* expressing) cells expressed ZsGreen and most ZsGreen+ cells co-expressed Nav1.8 confirming faithful and efficient expression of Dre-recombinase in sensory neurons (Figure S1B; Table S1A).

The second line is the intersectional Ai66 line (*Rosa26-rox-STOP-rox-lox-STOP-lox-tdTomato* mice), which expresses the fluorophore tdTomato from the ubiquitous Rosa26 locus (Madisen et al., 2015). Expression of tdTomato in this line is, however, not induced until the removal of two flanked STOP cassettes by Dre-recombinase and by Cre-recombinase (Figure 1B).

The third set of lines includes numerous Cre-expressing mouse lines, which we selected based on genetic markers identified by previous sequencing studies (Bai et al., 2019; Hockley et al., 2019; Kupari et al., 2019; Usoskin et al., 2015; Williams et al., 2016). By crossing these three sets of mouse lines, we generated triple transgenic mice that express tdTomato only in distinct sensory neurons, namely those expressing both Dre-recombinase (Nav1.8+) and Cre-recombinase (Figure 1B).

We first analyzed tdTomato expression in triple transgenic mice generated from a *Phox2b-Cre* line (Scott et al., 2011), which targets cells derived from epibranchial placodes, including vagal afferents, and a *Wnt1-Cre* line (Chai et al., 2000), which targets cells derived from the neural crest, including spinal afferents. In mice from the *Phox2b-Cre* line, we observed tdTomato expression in most NG neurons, which accurately expressed endogenous *Phox2b* as assessed by FISH (Figures 1C and S1C; Table S1B). No tdTomato+ cell bodies were found in DRG (Figure 1C; Table S1B). In mice from the *Wnt1-Cre* line, tdTomato was expressed in most DRG neurons (Figure 1D; Table S1B). No fluorescence was detected in NG neurons, whereas some tdTomato-containing cell bodies were observed nearby. Because jugular ganglia (JG) are adjunct to NG and derive, like DRG neurons, from the neural crest, we assessed the JG marker *Prdm12* (Kupari et al., 2019). FISH analysis confirmed that *Prdm12* was co-expressed in all tdTomato+ cell bodies in JG and DRG, in mice from the *Wnt1-Cre* line, but not in NG neurons in mice from the *Phox2b-Cre* line (Table S1B).

To determine the central projections of PHOX2B and WNT1 sensory neurons in triple transgenic mice, we assessed brainstem and spinal cord (Figure 1A). In mice from the *Phox2b-Cre* line, tdTomato-containing terminals densely innervated the NTS and the area postrema (AP) of the brainstem (Figure 1C), which receive innervation from vagal afferents (Berthoud and Neuhuber, 2000). Consistent with the absence of recombination in DRG, no PHOX2B terminals were detected in the spinal cord (Figure 1C). In mice from the *Wnt1-Cre* line, tdTomato-containing terminals were distributed in dorsal laminae at all levels of the spinal cord and in the spinal trigeminal nucleus (sp5) in the brainstem, but not in the NTS and AP (Figure 1D). Of note, the sp5 receives innervation from neural-crest-derived trigeminal ganglia (TG) sensory neurons. Collectively, these data demonstrate selective targeting of PHOX2B vagal afferents and WNT1 sensory neurons in triple transgenic mice derived from *Phox2b-Cre* and *Wnt1-Cre* mice, respectively.

Next, we obtained *Glp1r-ires-Cre* and *Gpr65-ires-Cre* mice (Williams et al., 2016) and generated tdTomato-expressing mice. We observed recombined cell bodies in NG in mice from both lines (Figures 1E, 1F, and S1C). We confirmed that the majority of tdTomato-containing NG neurons express endogenous *Glp1r* and *Gpr65* in mice derived from *Glp1r-ires-Cre* and *Gpr65-ires-Cre* mice, respectively (Figure S1C; Table S1B). To corroborate these findings, we assessed expression of the *Ccka* receptor (*Cckar*), the *neuropeptide Y 2 receptor* (*Npy2r*), and *Gpr65*, in mice from the *Glp1r-ires-Cre* line. Most tdTomato-containing NG neurons co-expressed *Cckar* and *Npy2r* but did not express *Gpr65*, confirming that GLP1R and GPR65 identify two non-overlapping vagal afferent populations (Figure S1D) (Egerod et al., 2018; Williams et al., 2016). The tdTomato-negative NG neurons with detectable *Glp1r* mRNA (Table S1B) could represent neurons without Nav1.8 expression (Bai et al., 2019; Kupari et al., 2019).

Analysis of tdTomato-containing axonal terminals revealed that GLP1R vagal afferents densely innervate the medial NTS and the AP, whereas GPR65 vagal afferent projections are mostly located just beneath the AP, medially to the NTS commissural zone, and fewer in the AP (Figures 1E and 1F). Importantly, no recombined cell bodies could be detected in DRG from both lines, and consistently, only very few, if any, tdTomato-containing axonal terminals were found in the spinal cord (Figures 1E and 1F). Thus, mice derived from *Glp1r-ires-Cre* and *Gpr65-ires-Cre* selectively target vagal afferents. The strikingly distinct NTS projections of GLP1R and GPR65 vagal afferents are consistent with previous analyses of these neurons (Bai et al., 2019; Williams et al., 2016), confirming specificity and efficiency of our intersectional genetic approach.

In addition, we generated tdTomato-expressing triple transgenic mice from *Trpv1-ires-Cre* (Cavanaugh et al., 2011), *Tac1-ires-Cre* (Harris et al., 2014), *Sst-ires-Cre* (Taniguchi et al., 2011), and *Vglut3-ires-Cre* (Tasic et al., 2018) mice. We confirmed accurate recombination in NG and DRG neurons in mice from all lines using FISH (Figures S1E–S1H; Table S1C). In mice targeting TRPV1, tachykinin precursor 1 (TAC1), and somatostatin (SST) sensory neurons, we observed tdTomato-containing cells in NG and DRG, and, consistent with their central projections, recombined terminals in brainstem and spinal cord (Figures S1E–S1G). TRPV1 terminals were densely distributed at all rostrocaudal levels of NTS, AP, sp5, and in the spinal dorsal horn (Figure S1E). This suggests that TRPV1 characterizes multiple vagal and spinal sensory neuron populations. TAC1 terminals were concentrated in the lateral NTS subnucleus, in the sp5, and distributed in the spinal dorsal horn (Figure S1F). SST terminals were located in the medial NTS, and in outer lamina II in the spinal cord (Figure S1G), revealing the distinct central projections of SST sensory neurons. In mice derived from the *Vglut3-ires-Cre* line, recombined cell bodies were located in DRG, and axonal terminals were observed in spinal lamina I and the innermost layer of lamina II (Figure S1H), reflecting VGLUT3 protein expression (Seal et al., 2009). VGLUT3 terminals were also observed in the sp5, while no cell bodies were found in NG, and no labeled terminals were found in the NTS and AP (Figure S1H), suggesting that VGLUT3 selectively marks sensory neurons of DRG and TG origin. Collectively, the above studies confirm accurate intersectional targeting of molecularly defined sensory neurons and identify their distinct central projections.

### Nav1.8 subpopulations innervate GI tract organs in distinct patterns

**Figure 2:**
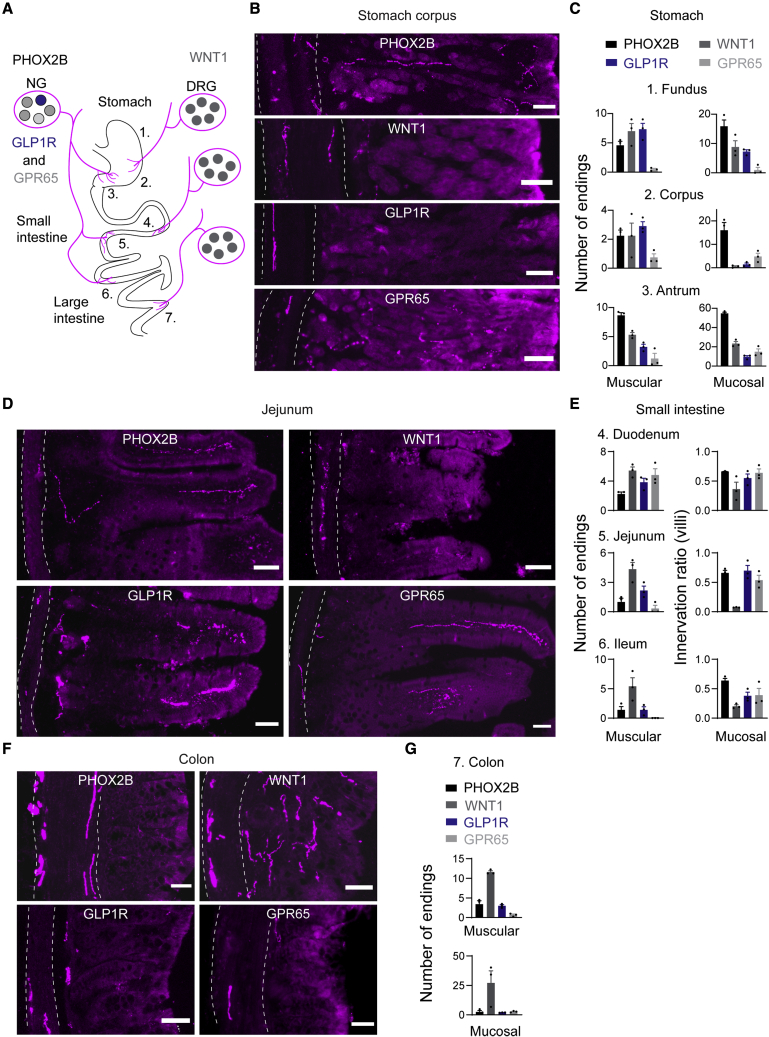
Intersectional mapping identifies the gut innervation patterns of distinct vagal and spinal afferents (A) Schematic of stomach, small intestine, and large intestine innervation by sensory neurons of NG and DRG origin. (B, D, and F) Representative images showing tdTomato-containing (magenta) endings in stomach corpus (B), jejunum (D), and colon (F). Scale bars represent 50 μm. Dashed lines indicate muscular layer. (C, E, and G) Quantification of tdTomato-containing mucosal and muscular terminal endings in triple transgenic mice derived from *Phox2b-Cre, Wnt1-Cre, Glp1r-ires-Cre*, and *Gpr65-ires-Cre* mice of the stomach (C), small intestine (E), and colon (G). Values are presented as mean ± SEM. See also Figure S2.

To determine the contribution of discrete sensory neuron populations in gut-brain communication, we next sought to reconstruct peripheral innervation of the GI tract organs in triple transgenic mice (Figure 2A). We first visualized tdTomato-containing terminals in mice from the *Phox2b-Cre* line, which target the majority of vagal afferents (Figure 1C), and quantified their innervation patterns by immunohistochemistry and imaging. In the stomach, we observed PHOX2B endings in muscular and mucosal layers, with the highest density in the antrum (Figures 2B, 2C, S2A, and S2B). Muscular endings in the stomach included intramuscular array (IMA) and intraganglionic laminar endings (IGLEs; Figure S2C), which are thought to serve as mechanoreceptors to detect tension and stretch (Berthoud et al., 2004). Along the entire length of the small intestine, PHOX2B endings were distributed, and quantitative analysis revealed that mucosal endings, which are putative chemosensory terminals (Berthoud et al., 2004), innervated approximately two-thirds of villi (Figures 2D and 2E). Innervation density of PHOX2B vagal afferents decreased beyond the ileum; in the colon, we observed significantly fewer endings in muscular layers and sparse innervation of crypts (Figures 2F and 2G). The dense mucosal and muscular innervation by vagal afferents further supports their key role in the relay of signals from upper GI tract organs (Berthoud et al., 2004), and acute feeding and glucoregulatory feedback control following food consumption (Clemmensen et al., 2017).

In mice derived from the *Glp1r-ires-Cre* and *Gpr65-ires-Cre* line, which selectively target vagal afferents (Figures 1E, 1F, and S1C; Table S1B), we observed tdTomato-containing endings in the stomach and small intestine (Figures 2B–2E, S2A, S2B, and S2D). Muscular endings from GLP1R vagal afferents were enriched in the stomach fundus and corpus, while only few mucosal endings were observed in these tissues (Figures 2B, 2C, and S2A). In contrast, stomach innervation by GPR65 vagal afferents was very sparse and mostly restricted to mucosal layers of corpus and antrum (Figures 2B, 2C, and S2B). In the small intestine, GLP1R and GPR65 vagal afferents densely innervated duodenal and jejunal villi, while fewer endings were found in the ileum (Figures 2D, 2E, and S2D). Muscular endings from both populations were also distributed in the small intestine, with the highest density of GLP1R endings in the duodenum (Figures 2E and S2D). In the colon, we observed only very few GLP1R and GPR65 endings (Figures 2F and 2G). Thus, GLP1R and GPR65 vagal afferents display distinct innervation patterns of stomach and small intestine, with terminals following previously described endings (Bai et al., 2019; Berthoud et al., 2004; Gautron et al., 2011; Williams et al., 2016).

In mice from the *Wnt1-Cre* line, which target spinal afferents but avoid vagal afferents (Figure 1D), we observed tdTomato-containing endings throughout the gut (Figures 2B–2G and S2A–S2D). Innervation of the stomach and small intestine was, however, sparser, as compared with vagal afferent innervation, and endings were more frequently detected in muscular layers (Figures 2B–2E and S2A–S2D). In the stomach, dense innervation by WNT1 endings was observed in the fundus and antrum (Figures 2C, S2A, and S2B). In the small intestine, we observed dramatically fewer WNT1 endings, as compared with vagal afferents, especially in the jejunum, where only ∼10% of villi contained tdTomato-positive fibers (Figures 2D, 2E, and S2D). Interestingly, beyond the jejunum, density of muscular endings increased and quantitative analysis revealed that colon innervation of WNT1 spinal afferents was more than 2-fold as compared with vagal afferents (Figures 2F and 2G). Furthermore, innervation of colon crypts was ∼10-fold more as compared with PHOX2B innervation (Figures 2F and 2G). Thus, dense innervation of ileum and colon are defining characteristics of spinal afferents. The mucosal endings in the colon likely correspond to lumbar DRG neurons, as revealed previously (Brierley et al., 2018; Green and Dockray, 1988; Hockley et al., 2019; Spencer et al., 2014). Notably, GLP1R and GPR65 vagal afferents are distinct from WNT1 neurons, as they do not express *Prdm12* but correspond to the PHOX2B population of vagal afferents (Kupari et al., 2019).

In addition to gut-brain communication, sensory neurons of vagal and spinal origin transmit information from other abdominal organs (Cervero, 1994). To probe whether the above four populations contribute to this communication, we analyzed the extent of tdTomato-containing ending innervation in supra- and subdiaphragmatic organs. We found that PHOX2B vagal afferents are sparsely distributed in trachea, heart, lung, and kidney, whereas dense innervation was observed in liver and gallbladder (Figures S2E–S2G). WNT1 spinal afferents, on the other hand, were enriched in trachea and heart, while only sparse innervation of other abdominal organs could be detected (Figures S2E–S2G). Importantly, GLP1R and GPR65 vagal afferents provide no, or only very-limited, innervation of non-gut abdominal organs (Figures S2E–S2G) that is detectable using this intersectional approach. The sparsity of supradiaphragmatic organ innervation by vagal afferents, as compared with previous tracing studies (Chang et al., 2015; Prescott et al., 2020), could reflect that our genetic approach selectively targets neurons expressing Nav1.8, which is enriched in vagal afferents innervating subdiaphragmatic organs (Bai et al., 2019).

TRPV1 sensory neurons have been implicated in diverse cellular and physiological processes, including the intestinal response to infection (Lai et al., 2020). Consistent with our finding that mice from the *Trpv1-ires-Cre* line target large proportions of vagal and spinal afferents (Figure S1E), we observed numerous tdTomato-containing muscular and mucosal endings in the stomach and duodenum (Figures S2H and S2I). In contrast to this widespread labeling, TAC1 sensory neuron endings innervated only muscular layers of stomach and duodenum (Figures S2H and S2I). Since TAC1 largely targets DRG neurons (Figure S1F), innervation of these organs likely corresponds to spinal afferents, as suggested previously (Spencer et al., 2016). SST sensory neuron endings were observed in muscular layer in the stomach (Figure S2H), which could correspond to SST vagal afferent innervation (Bai et al., 2019). Interestingly, terminal endings of VGLUT3 sensory neurons, which avoid vagal afferents but target spinal afferents (Figure S1H), were not observed in the stomach and duodenum (Figures S2H and S2I). Thus, taken together with the above PHOX2B and WNT1 mapping studies, these findings demonstrate that muscular endings of spinal afferents, including TAC1, but not VGLUT3, innervate stomach and duodenum.

### GLP1R and GPR65 vagal afferents engage different feeding and glucoregulatory neurocircuits

**Figure 3:**
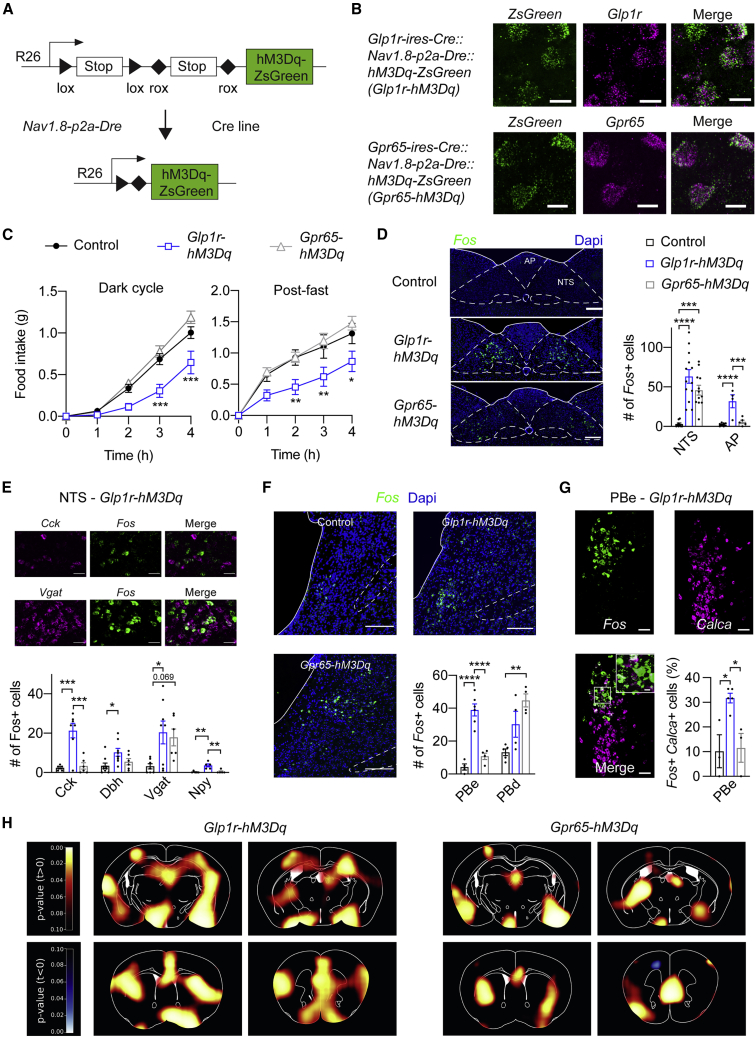
Selective stimulation of gut-innervating vagal afferents alters feeding and modulates neuronal activity in distinct brain regions (A) Breeding schematic and schematic diagram of the Rosa-26-targeting vector allowing Cre-/Dre-dependent expression of hM3Dq-ZsGreen. Excision of lox-flanked and rox-flanked stop cassettes lead to hM3Dq-ZsGreen expression. (B) *hM3Dq-ZsGreen* and endogenous *Glp1r* and *Gpr65* mRNA expression in NG from triple transgenic mice derived from *Glp1r-ires-Cre* and *Gpr65-ires-Cre* mice. Scale bars represent 20 μm. (C) Effects of hM3Dq-induced stimulation of GLP1R or GPR65 vagal afferents on dark-cycle feeding (left) and on (post-fast) refeeding after 16 h of fasting (right). Mice per group, n = 8–19. (D–G) *Fos* expression in NTS (D and E) and PB (F and G) upon chemogenetic stimulation of GLP1R and GPR65 vagal afferents assessed by FISH. Acutely stimulating GLP1R vagal afferents induces *Fos* in the PBe (F and G) while stimulating GPR65 vagal afferents induces *Fos* in a discrete region of the PBd (F). Scale bars represent 100 μm (NTS) or 200 μm (PBN). Analyzed sections per group, n = 3–13. (H) Brain activation pattern upon stimulation of the two subtypes as assessed by [^18^F]FDG PET (p values from voxelwise t test are indicated by color bar). In all experiments, triple transgenic mice and littermate controls were injected with CNO. Mice are from multiple litters. Statistical significance was assessed by two-way mixed effects ANOVA (C) with Dunnett’s test for multiple comparisons, or ordinary one-way ANOVA with Tukey’s test for multiple comparisons (D–G). Significant results are indicated by ^∗^p ≤ 0.05, ^∗∗^p ≤ 0.01, ^∗∗∗^p ≤ 0.001, and ^∗∗∗∗^p < 0.0001. Values are presented as mean ± SEM. See also Figure S3.

Having established the selective gut innervation by GLP1R and GPR65 vagal afferents, we next aimed to obtain a precise understanding of their feeding and glucoregulatory function. To directly probe the sufficiency of these neurons in the control of food intake and glucose metabolism, we intersectionally expressed the chemogenetic receptor construct hM3Dq. We crossed mice allowing expression of hM3Dq-ZsGreen in Dre- and Cre-recombinase expressing cells (*Rosa26-lox-STOP-lox-rox-STOP-rox-hM3Dq-ZsGreen* mice; (Biglari et al., 2021)) with *Nav1.8-p2a-Dre* mice and Cre-expressing mice to yield triple transgenic animals and control littermates (Figure 3A). We confirmed expression of the fused fluorophore *ZsGreen* in *Glp1r* and *Gpr65* expressing cells in NG, in mice derived from *Glp1r-ires-Cre* and *Gpr65-ires-Cre* mice, respectively, using FISH (Figures 3B and S3A). No transgene expression was found in DRG (Figure S3A), demonstrating accurate intersectional targeting of hM3Dq-ZsGreen to these two non-overlapping vagal afferent populations. We observed more ZsGreen expressing NG cells in mice from the *Glp1r-ires-Cre* line (Figure S3A), consistent with the relatively higher number of GLP1R versus GPR65 vagal afferents (Bai et al., 2019; Kupari et al., 2019; Williams et al., 2016).

In hM3Dq-expressing mice from the *Glp1r-ires-Cre* line, clozapine-N-oxide (CNO) administration reduced food intake during the dark cycle when compared with littermate controls (Figure 3C). Food intake in calorically depleted mice that were fasted for 16 h was also reduced (Figure 3C), whereas feeding during the light cycle was not affected (Figure S3B). In hM3Dq-expressing mice from the *Gpr65-ires-Cre* line, however, CNO administration failed to significantly alter food intake during the dark cycle, the light cycle, and after fasting (Figures 3C and S3B). Thus, acute activation of GLP1R, but not GPR65, vagal afferents is sufficient to reduce feeding even in the context of caloric deprivation.

The NTS is the central target of vagal afferents (Figure 1A), and numerous distinct neuronal populations in this brainstem region have been implicated in food intake regulation (Aklan et al., 2020; Andermann and Lowell, 2017; D'Agostino et al., 2016; Gaykema et al., 2017; Ludwig et al., 2021; Roman et al., 2016). Given this, we assessed whether and which NTS neurons are the downstream effectors of GLP1R and GPR65 vagal afferents. Specifically, we examined expression of *Fos* and numerous neuronal markers (*Cck*, dopamine beta-hydroxylase [*Dbh*], *Vgat* [*Slc32a*], *Npy*, and glucagon [*Gcg*]) in hM3Dq-expressing mice following CNO administration using FISH. Stimulation of GLP1R vagal afferents caused profound increases of *Fos* in the NTS (Figures 3D and 3E). Additionally, *Fos* in the AP, which is innervated by GLP1R vagal afferents (Figure 1E; Bai et al., 2019; Williams et al., 2016), was increased (Figure 3D). Stimulating GPR65 vagal afferents also increased *Fos* in the NTS, but to a lesser extent (Figures 3D and 3E). FISH analysis revealed that GLP1R vagal afferent stimulation was more effective in activating *Cck*-, *Dbh*-, and *Npy*-expressing neurons, as compared with GPR65 vagal afferent stimulation, while no significant difference could be detected in *Gcg*-expressing cells (Figures 3E, S3C, and S3DA). Interestingly, stimulating either vagal afferent population activated a similar proportion of GABAergic (*Vgat*-expressing) neurons in the NTS (Figures 3E and S3C).

We next tested whether activation of GLP1R and GPR65 vagal afferents increases neural activity in the lateral parabrachial nucleus (PB). This possibility is of interest because NTS and AP neurons project to and synaptically engage PB neurons that control feeding behavior (Campos et al., 2016; Carter et al., 2013; Han et al., 2018; Kim et al., 2020; Roman et al., 2016; Zhang et al., 2021). We found that GLP1R vagal afferent stimulation increased neuronal activity in the external lateral part of the PB (PBe; Figures 3F and 3G). Using FISH, we revealed that activated PBe neurons express *Calca*, which encodes calcitonin gene-related peptide (CGRP; Figure 3G). This is of particular interest because CGRP expressing PBe (PBe^CGRP^) neurons receive strong synaptic input from CCK-expressing NTS neurons (Roman et al., 2016) and mediate the satiating effects of gut-derived signals (Campos et al., 2016; Carter et al., 2013). GLP1R vagal afferent stimulation also increased neural activity in the dorsal part of the PB (PBd), but to a lesser extent (Figures 3F and 3G). Remarkably, although stimulating GPR65 vagal afferents increased neuronal activity in the PB, it failed to activate PBe^CGRP^ neurons (Figures 3F and S3E). Fos expression was, however, more robustly increased in the PBd (Figure 3F), which we later discover is more selective in CCK-expressing neurons (Figure 5E). Thus, our data suggest that GLP1R vagal afferents, whose activation reduces feeding, selectively control PBe^CGRP^ neuron activity.

In addition, we determined brain sites downstream of GLP1R and GPR65 vagal afferents by employing positron emission tomography (PET) with 2-deoxy-2-[^18^F]fluoro-D-glucose ([18F]FDG). Consistent with the increases in Fos in the AP/NTS, stimulation of either population induced a significant activation pattern in this brainstem region (Figure S3F). Stimulating GLP1R vagal afferents also caused an activation in the PB, the bed nucleus of the striae terminalis (BNST), medial basal hypothalamus (MBH), and the supraoptic nucleus (SON) of the hypothalamus (Figures 3H and S3F). Activation was also found in a number of regions rostral to the NTS—such as the ventral tegmental area (VTA), the paraventricular thalamus (PVT), the basolateral amygdala (BLA), the insular cortex (IC), and the dorsal striatum (DS; Figures 3H and S3F). Stimulation of GPR65 vagal afferents caused a significant activation in MBH, VTA, PVT, and BLA, whereas no activation was found in BNST, SON, IC, and DS (Figures 3H and S3F). Thus, stimulation of these two non-overlapping, gut-innervating vagal afferents causes distinct neuronal activity patterns demonstrating that they engage different neurocircuits.

### GLP1R vagal afferents relay gut-derived anorexigenic signals

**Figure 4:**
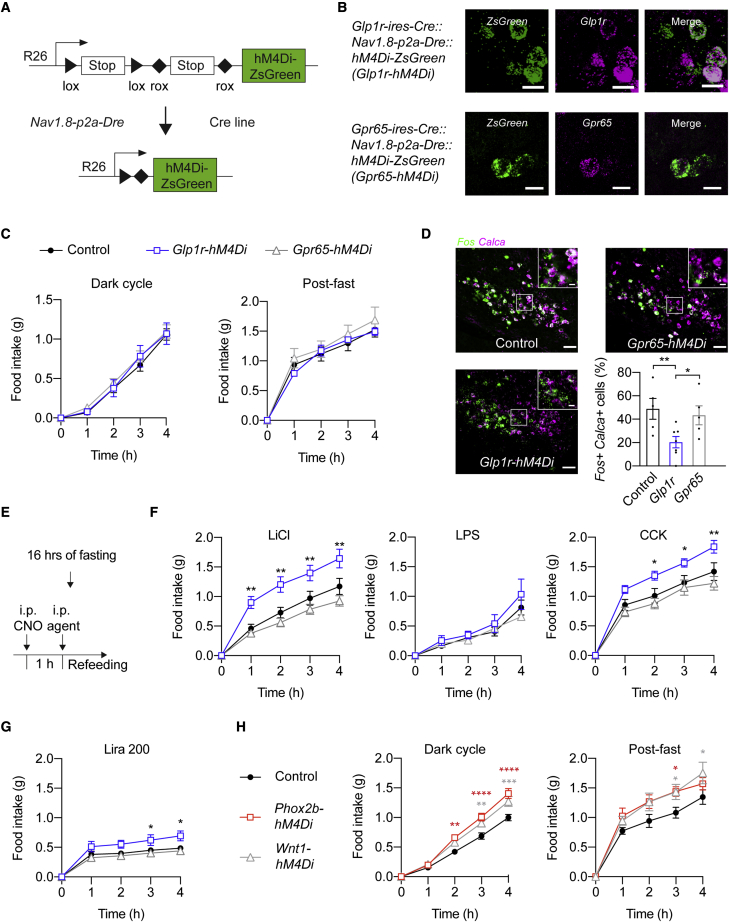
GLP1R vagal afferent activity contributes to LiCl- and CCK-induced anorexia (A) Schematic diagram of the Rosa-26-targeting vector allowing Cre-/Dre-dependent expression of hM4Di-ZsGreen. (B) *hM4Di-ZsGreen* and endogenous *Glp1r* and *Gpr65* expression in NG in mice derived from *Glp1r-ires-Cre* and *Gpr65-ires-Cre* mice, respectively. Scale bars represent 20 μm. (C) Effects of hM4Di-induced inhibition on dark-cycle feeding (left) and on refeeding after 16 h fasting (right). Mice per group, n = 5–16. (D) Representative histological images and analysis of *Fos* expression in PBe^CGRP^ neurons in hM4Di-expression mice following LiCl injection assessed by FISH. *Calca* encodes CGRP. Scale bars represent 100 μm. (E) Schematic of the experimental protocol used for determining the anorexigenic effects of different agents. (F and G) Effects of CNO/hM4Di-induced inhibition of GLP1R or GPR65 vagal afferents on refeeding after administration of LiCl, LPS, CCK (F), or a high dose of liraglutide (200 mg/kg; Lira 200, G). Mice per group, n = 4–19. (H) Effects of hM4Di-induced inhibition of PHOX2B or WNT1 sensory neurons on dark-cycle feeding (left) and on refeeding after 16-h fasting (right). Mice per group, n = 7–16. In all experiments, triple transgenic mice and littermate controls were injected with CNO. Mice are from multiple litters. Statistical significance was assessed by two-way mixed-effects ANOVA (C, F, G, and H) or one-way ANOVA (D) with Dunnett’s test for multiple comparisons. Significant results are indicated by ^∗^p ≤ 0.05, ^∗∗^p ≤ 0.01, ^∗∗∗^p ≤ 0.001, and ^∗∗∗∗^p < 0.0001. Values are presented as mean ± SEM. See also Figure S4.

To investigate the necessity of GLP1R and GPR65 vagal afferents in the regulation of feeding, we generated a Rosa26-based mouse line for Cre-/Dre-dependent expression of the inhibitory chemogenetic receptor hM4Di (*Rosa26-lox-STOP-lox-rox-STOP-rox-hM4Di-ZsGreen*; Figure 4A; STAR methods). Triple transgenic mice expressing hM4Di in sensory neurons were generated by crossing these mice with *Nav1.8-p2a-Dre* and Cre-expressing mice (Figure 4A). We confirmed that CNO inhibited hM4Di-ZsGreen-expressing sensory neurons as assessed by whole-cell patch-clamp recordings (Figure S4A). In mice derived from *Glp1r-ires-Cre* and *Gpr65-ires-Cre* mice, we observed *ZsGreen* expression in *Glp1r*- and *Gpr65*-expressing cells in the NG, respectively, whereas no expression was found in DRG (Figures 4B and S4B), confirming accurate intersectional recombination in vagal afferents.

CNO administration in hM4Di-expressing mice from both *Glp1r-ires-Cre* and *Gpr65-ires-Cre* lines failed to affect dark-cycle feeding and refeeding after a 16-h fast (Figure 4C). Light-cycle feeding was also not acutely altered (i.e., during the first 3 h after CNO injection) by inhibition of either neuronal population (Figure S4C). However, 4 h after CNO injection mice from the *Glp1r-ires-Cre* line responded with a small increase in food intake (Figure S4C). Thus, although stimulating GLP1R vagal afferents rapidly and profoundly reduces food intake (Figure 3C; Bai et al., 2019), their activity is not necessary for the acute regulation of feeding.

Vagal afferents relay gut-derived information to the brain that causes appetite suppression. As a first step to determine whether transmission of gut-borne anorexigenic signals requires GLP1R or GPR65 vagal afferent activity, we asked whether their selective inhibition blunts Fos expression in PBe^CGRP^ after injection of lithium chloride (LiCl; Figure 4D) (Carter et al., 2013). Chemogenetic inhibition of GLP1R vagal afferents profoundly reduced PBe^CGRP^ neuron activation after injection of LiCl (Figure 4D). We therefore predicted that inhibition of GLP1R vagal afferents, similar to inhibition of PBe^CGRP^ neurons (Carter et al., 2013), would reduce LiCl-induced anorexia. Consistent with this hypothesis, GLP1R vagal afferent inhibition ameliorated the reduction of food intake after LiCl injection when compared with littermate controls (Figures 4F and S4D). Inhibition of GPR65 vagal afferents, however, failed to reduce PBe^CGRP^ neuron activity and caused no alteration in food intake reduction following LiCl injection (Figures 4F and S4D). Based on previous findings demonstrating that PBe^CGRP^ neuron activity contributes to anorexigenic effects of lipopolysaccharide (LPS; Carter et al., 2013), we probed whether inhibition of GLP1R or GPR65 vagal afferents would blunt LPS-induced anorexia. Chemogenetic inhibition of GLP1R and GPR65 vagal afferents failed, however, to alter the suppression of feeding after LPS injection (Figure 4F).

Next, we assessed whether GLP1R and GPR65 vagal afferents are involved in mediating the anorexigenic effects of the gut hormones CCK and GLP-1. This possibility is of interest because PBe^CGRP^ neurons are also involved in transmitting this information (Campos et al., 2016). Specifically, we examined the consequences of injecting CCK-8 or the GLP1R agonist liraglutide on feeding in hM4Di-expressing mice. We found that chemogenetic inhibition of GLP1R vagal afferents abolished the reduction of feeding after injection of CCK (Figure 4F). Food intake reduction by liraglutide was, however, only slightly ameliorated (Figures 4G and S4C). Inhibition of GPR65 vagal afferents failed to alter food intake reduction after injection of CCK and liraglutide (Figures 4F, 4G, and S4D). Collectively, these studies demonstrate that GLP1R vagal afferents selectively mediate the anorexigenic effects of LiCl and CCK.

Given that inhibition of GLP1R and GPR65 vagal afferents failed to acutely increase steady-state feeding, we hypothesized that other sensory neuron populations are responsible for the observed changes in food intake upon surgical or pharmacological removal of vagal and spinal afferents (De Vadder et al., 2014; Duraffourd et al., 2012; Ritter and Ladenheim, 1985; van de Wall et al., 2005; Walls et al., 1995). Thus, we examined whether chemogenetic inhibition of PHOX2B sensory neurons, which mark most vagal afferents, or WNT1 sensory neurons, which mark most spinal afferents (Figures 1C, 1D, and S4E; Kupari et al., 2019; Scott et al., 2011), alters feeding. We found that inhibition of PHOX2B and WNT1 sensory neurons increased food intake during the dark cycle and after fasting (Figure 4H). While PHOX2B and WNT1 sensory neurons transmit information from numerous organs, including abdominal organs (Figures S2A–S2F), many of which could promote anorexia, the observed increases in food intake upon chemogenetically silencing these cells suggest that subsets of vagal and spinal afferents are involved in acutely regulating steady-state feeding.

### Gut-innervating vagal afferents differently control glucose tolerance and hepatic glucose production

**Figure 5:**
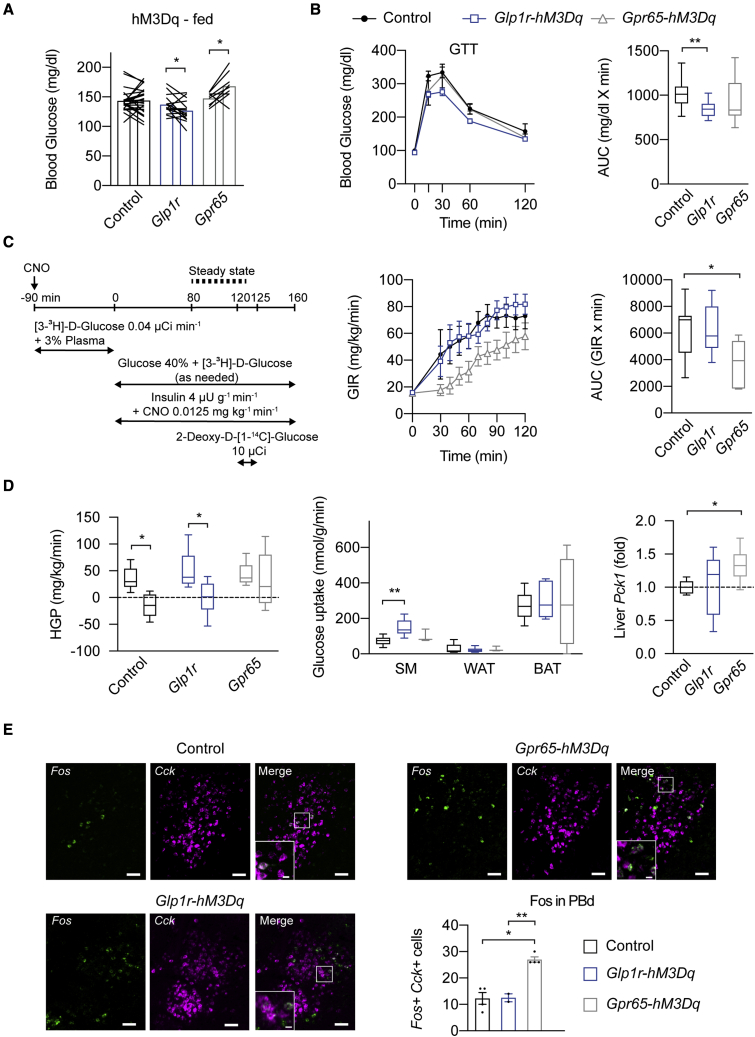
Acute stimulation of GLP1R and GPR65 vagal afferents differently affects glucose homeostasis (A) Effects of hM3Dq-induced stimulation of GLP1R or GPR65 vagal afferents on blood glucose levels in fed mice. Mice per group, n = 9–24. (B) Glucose tolerance in hM3Dq-expressing mice and littermate controls 1 h after CNO administration. Mice per group n = 9–21. (C) Schematic (left) of the experimental protocol for euglycemic-hyperinsulinemic clamp studies. Glucose infusion rate (GIR; right) during clamp studies in hM3Dq-expressing mice and littermate controls. Mice per group, n = 7–9. (D) HGP during basal and steady state of the clamp. Glucose uptake in skeletal muscle (SM), white adipose tissue (WAT), and brown adipose tissue (BAT). Hepatic *Pck1* gene expression after clamp. Mice per group, n = 6–8. (E) Representative histological images and analysis of *Fos* expression in the PBd^CCK^ neurons in hM3Dq-expression mice following CNO injection. Scale bars represent 100 μm. In all experiments, triple transgenic mice and littermate controls were injected with CNO. Mice are from multiple litters. Statistical significance was assessed by two-tailed paired Student’s t test (A and D, left), or ordinary one-way ANOVA with Dunnett’s (B, C, and D, middle, right) or Tukey’s (E) test for multiple comparisons. Significant results are indicated by ^∗^p ≤ 0.05 and ^∗∗^p ≤ 0.01. Values are presented as mean ± SEM. See also Figure S5.

In addition to feeding, gut-innervating sensory neurons have been implicated in the regulation of glucose homeostasis (Clemmensen et al., 2017). Given this, we tested the acute glucoregulatory function of GLP1R and GPR65 vagal afferents. We first determined how chemogenetically stimulating these neurons affects blood glucose levels in fed animals. These studies were performed in absence of food. We found that stimulation of GLP1R vagal afferents decreased blood glucose levels in fed animals (Figure 5A). Blood glucose levels were, however, increased upon stimulation of GPR65 vagal afferents (Figure 5A). No changes in blood glucose levels were observed in fasted mice from both lines (Figure S5A). When we assessed serum insulin, we found that stimulation of GLP1R vagal afferents caused a slight, yet not significant, increase in insulin levels (Figure S5B). Serum glucagon and corticosterone levels were unaffected in mice from both lines (Figure S5B). We next performed glucose and insulin tolerance tests (GTTs and ITTs) in triple transgenic mice and found that stimulation of GLP1R vagal afferents improved glucose tolerance, while no effects were observed during stimulation of GPR65 vagal afferents (Figure 5B). Insulin sensitivity during ITTs was not affected in mice from either line (Figure S5C).

To further define how these two gut-innervating vagal afferent populations regulate peripheral glucose metabolism, we performed euglycemic-hyperinsulinemic clamp studies (Figure 5C). Stimulation of GLP1R vagal afferents failed to affect the glucose infusion rate (GIR) required to maintain euglycemia when compared with littermate controls (Figure 5C). Insulin’s ability to promote glucose uptake in skeletal muscle was, however, significantly increased in steady state (Figure 5D). In contrast, stimulating GPR65 vagal afferents induced a decrease in the GIR as compared with littermate controls (Figure 5C). Importantly, when we assessed hepatic glucose production (HGP), no differences in baseline and insulin-suppressed rate of HGP upon stimulation of GPR65 vagal afferents was observed (Figure 5D). Furthermore, under clamp conditions, stimulation of GPR65 vagal afferents caused an increase in hepatic mRNA levels of the gluconeogenic gene phosphoenolpyruvate carboxykinase (*Pck1*; Figure 5D). No significant changes of serum corticosterone and hepatic mRNA levels of glucose 6-phosphatase were observed in mice from both lines under clamp conditions (Figure S5D). Thus, acutely activating GLP1R vagal afferents improves glucose tolerance by increasing glucose uptake in skeletal muscles. In contrast, activation of GPR65 vagal afferents increases HGP by increasing *Pck1*.

Given that HGP is stimulated by neural counter-regulatory responses (CRR) to hypoglycemia (Stanley et al., 2019), we hypothesized that GPR65 vagal afferents activate the underlying neurocircuits. Based on our finding that Fos in the PBd was increased by GPR65 vagal afferent activation (Figure 3F), we postulated that they activated CCK-expressing (PBd^CCK^) neurons, which mediate CRR (Flak et al., 2014; Garfield et al., 2014). In support of this hypothesis, stimulating GPR65 vagal afferents activated PBd^CCK^ neurons as assessed using Fos (Figure 5E). In contrast, GLP1R vagal afferent activation failed to activate PBd^CCK^ neurons (Figure 5E).

**Figure 6:**
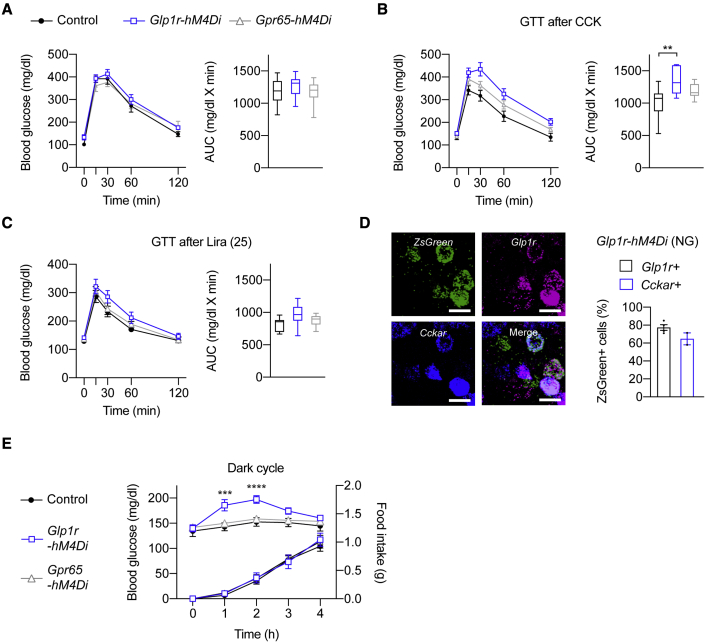
Selective inactivation of GLP1R vagal afferents disrupts glycemic control during feeding (A–C) Effects of hM4Di-induced inhibition of GLP1R or GPR65 vagal afferents on glucose tolerance during GTTs. CCK (B) or liraglutide (C) were administered 15 min before glucose injections. Mice per group, n = 7–10. (D) Representative images (left) and analysis (right) of endogenous *Glp1r* and *Cckar* expression in hM4Di-ZsGreen expressing NG neurons from *Glp1r-hM4Di* mice. Scale bars represent 20 μm. (E) Effects of hM4Di-induced inhibition of GLP1R or GPR65 vagal afferents on blood glucose levels during dark-cycle feeding. Mice per group, n = 8–10. In all experiments, triple transgenic mice and littermate controls were injected with CNO. Mice are from multiple litters. Statistical significance was assessed by ordinary one-way ANOVA with Dunnett’s test for multiple comparisons (A–C), or two-way mixed effects ANOVA with Dunnett’s test for multiple comparisons (E). Significant results are indicated by ^∗∗^p ≤ 0.01, ^∗∗∗^p ≤ 0.001, and ^∗∗∗∗^p < 0.0001. Values are presented as mean ± SEM. See also Figure S6.

To test the hypothesis that GLP1R and GPR65 vagal afferents participate in the physiological control of glucose homeostasis, we next employed hM4Di-expressing mice. We found that chemogenetic inhibition of both GLP1R and GPR65 vagal afferents failed to significantly alter GTTs and ITTs (Figures 6A and S6A). This suggests that activity of these neurons by themselves is not necessary for the regulation of glucose tolerance and insulin sensitivity. We next considered the possibility that GLP1R and GPR65 vagal afferents participate in the glucoregulatory actions of enteroendocrine hormones (Steinert et al., 2017). Our prediction based on the known glucoregulatory function of CCK and GLP-1 was that activity of gut-innervating vagal afferents mediates the glucose tolerance improving effects of these hormones. To test this hypothesis, we performed GTTs after injection of CCK-8 or liraglutide. CNO/hM4Di-induced inhibition of GLP1R vagal afferents abolished the improvement of glucose tolerance after CCK-8 injection (Figure 6B). Improved glucose tolerance after injection of a low (25 mg/kg) and a high (200 mg/kg) dose of liraglutide was, however, not reversed by inhibition of GLP1R vagal afferents (Figures 6C and S6B). These findings raise the possibility that CCK, but not GLP-1, directly activates GLP1R vagal afferents, which improves glucose tolerance. Consistent with this, FISH analysis revealed that the vast majority of hM4Di-expressing NG neurons in mice from the *Glp1r-ires-Cre* line co-express *Cckar* and *Glp1r* (Figure 6D). Inhibition of GPR65 vagal afferents slightly, yet not significantly, abolished the improved glucose tolerance after CCK-8 injection but did not affect glucose tolerance improvement after injection of liraglutide (Figures 6B, 6C, and S6B).

To further dissect the physiological relevance of GLP1R and GPR65 vagal afferent activity in glucose homeostasis regulation, we measured changes in blood glucose levels at the onset of the dark cycle when mice naturally engage in feeding. We found that inhibition of GLP1R vagal afferents resulted in an increase of blood glucose levels in the first 2 h (Figure 6E). Notably, and consistent with our prior findings (Figure 4C), food intake was not altered by acute inhibition of GLP1R vagal afferents (Figure 6E). Inhibition of GPR65 vagal afferents failed to affect blood glucose levels during dark-cycle feeding (Figure 6E). Together, these findings demonstrate that activity of gut-innervating GLP1R vagal afferents, which express the *Cckar* and directly respond to CCK (Williams et al., 2016), is of particular importance for the control of glycemia during feeding.

## Discussion

Sensory neurons densely innervate the different organs of the GI tract, and extensive surgical and pharmacological lesion studies have demonstrated that these cells are crucial for relaying food-derived signals from the gut to the brain (Clemmensen et al., 2017; Kim et al., 2018; Schwartz et al., 2000; Soty et al., 2017). However, the identity of the key vagal and spinal afferents that are involved in the regulation of feeding and glucose homeostasis has remained largely unclear. Recent single-cell sequencing studies have cataloged sensory neurons and determined gut-innervating populations (Bai et al., 2019; Hockley et al., 2019; Kupari et al., 2019; Usoskin et al., 2015). Here, we developed a Cre/Dre-dependent intersectional approach to facilitate genetic entry into sensory neurons, as a step toward assessing the discrete feeding and glucoregulatory function(s) of these cells and the underlying neurocircuits. This approach allowed for highly specific and efficient targeting defined populations of Nav1.8-expressing sensory neurons and anatomical reconstruction of their peripheral and central projections. Further, it allowed for chemogenetically manipulating their activity—and thereby serves as a platform for gain- and loss-of-function studies interrogating sensory neural circuits, including gut-brain communication.

We comprehensively interrogated the GI tract innervation pattern of numerous Nav1.8-expressing sensory neuron subpopulations and found that vagal and spinal afferents possess distinct but partly overlapping innervation pattern of the GI tract organs. We demonstrate that vagal afferents densely innervate muscular and mucosal layers of the stomach and upper small intestine, while spinal afferent endings in these organs are sparser and primarily located in muscular layers. In the lower gut, however, spinal afferent innervation is dramatically denser and particularly concentrated in colon crypts. These tissue-specific innervation patterns are consistent with previous analyses (Berthoud et al., 2004), possibly aligning with parallel transmission of gut-derived signals via vagal and spinal pathways. In agreement with previous studies (Bai et al., 2019; Williams et al., 2016), we found that GLP1R and GPR65 vagal afferents selectively innervate the stomach and intestine and have topographically disparate projections in the NTS and AP, pointing further to the relevance of these neurons in sensing disparate food-derived signals and engaging different downstream neural circuits in the brain.

Because stimulation of gut-innervating sensory neurons after a meal has been implicated in promoting satiation, we assessed food intake in response to manipulations of GLP1R or GPR65 vagal afferents through hM3Dq and hM4Di chemogenetic receptors. Interestingly, we found no acute changes in feeding following chemogenetic inhibition of either population. The lack of changes upon GLP1R vagal afferent inhibition was especially unexpected, given that we (Figure 3C) and others (Bai et al., 2019) have found that acutely stimulating this gut-innervating population potently reduces food intake. This discrepancy could reflect that their activation decreases appetite only under certain conditions. Indeed, we demonstrate that GLP1R vagal afferent inhibition selectively ameliorates the appetite suppressing action of the malaise-inducing agent LiCl and the enteroendocrine hormone CCK. Transmission of this information to ascending brain sites that regulate feeding behavior likely involves DBH- and CCK-expressing NTS neurons and downstream PBe^CGRP^ neurons (Campos et al., 2016; Carter et al., 2013; Roman et al., 2016) as determined by our Fos mapping studies. Importantly, the ability of the visceral stressor LPS to reduce feeding, which also requires PBe^CGRP^ neuron activation (Carter et al., 2013), was unaffected by inhibition of GLP1R vagal afferents. This raises the distinct possibility that gut-innervating GLP1R vagal afferents are not involved in detecting inflammatory signals. In striking contrast, activity of GPR65 vagal afferents, despite their dense innervation of the small intestine (Figure 2; Bai et al., 2019; Williams et al., 2016), is dispensable for transmission of anorexigenic, gut-derived, stimuli, and food intake regulation.

Nevertheless, using a broad marker to inhibit PHOX2B-expressing sensory neurons, we confirm the necessity of vagal afferents, as a whole, in acutely promoting satiation under basal feeding conditions. The sensory neuron subtype(s) responsible for these effects are currently unknown but could be vagal afferents expressing the oxytocin receptor, which, when artificially stimulated, potently suppress feeding (Bai et al., 2019). Interestingly, we found that inhibition of WNT1 sensory neurons similarly increased feeding, further suggesting the importance of the spinal pathway in the regulation of food intake, which often is considered as redundant. Notably, spinal afferents innervate GI tract organs, and their chronic ablation results in profound deregulation of energy homeostasis (Brierley et al., 2018; De Vadder et al., 2014; Duraffourd et al., 2012; Green and Dockray, 1988; Hockley et al., 2019; Spencer et al., 2014). Additionally, spinal gut-brain transmission upon intestinal glucose administration has recently been identified as required for the acute regulation of agouti-related peptide-expressing neurons (Goldstein et al., 2021), a hypothalamic neuron population essential for the control of hunger (Andermann and Lowell, 2017). However, because our genetic approach using *Phox2b-Cre* and *Wnt1-Cre* mice not only targets GI tract innervating sensory neurons, but also those innervating other organs (Figure S2), further cell-type-specific studies are required to identify the responsible spinal afferent population(s).

In addition to feeding, gut-innervating sensory neurons have been implicated in the regulation of peripheral glucose metabolism (Clemmensen et al., 2017; Dranse et al., 2018; Duca et al., 2015; Wang et al., 2008), yet the populations involved and the downstream neurocircuits they engage remained largely unclear. Thus, we systemically investigated the glucoregulatory function of GLP1R and GPR65 vagal afferents. As evidenced by our GTT and clamp studies, GLP1R vagal afferent activation is sufficient to improve glucose tolerance, which results from increased glucose uptake in skeletal muscles. Building on these findings, we assessed glucose concentration during basal feeding and demonstrate that selective inhibition of GLP1R vagal afferents increases blood glucose levels without affecting food intake. Since their activity was necessary for CCK-induced improvement of glucose tolerance, we suspect that CCK released from enteroendocrine cells during food consumption (Steinert et al., 2017) is crucial for the glucoregulatory action of GLP1R vagal afferents. Consistent with the latter, GLP1R vagal afferents express the Cckar, and CCK profoundly stimulates their activity as determined by previous *in vivo* imaging studies (Williams et al., 2016). Thus, our data demonstrate that GLP1R vagal afferent activity plays a crucial role in the control of blood glucose levels during feeding, but not food intake. Since high-fat diet feeding attenuates CCK-induced activation of vagal afferents as well as responses in downstream brain regions (Covasa et al., 2000; Troy et al., 2016), alterations in GLP1R vagal afferent control of meal-related glycemia may also provide a neural mechanism underlying impaired glycemic control in obesity (Steinert et al., 2017).

Our studies additionally unveiled that gut-innervating GPR65 vagal afferent activity is sufficient to increase blood glucose levels, which is probably mediated by increased HGP. Several key data provide strong evidence that the engagement of different downstream neural circuits explain these strikingly disparate glucoregulatory effects. As determined by our, as well as previous, tracing studies, GLP1R and GPR65 vagal afferents possess different projection fields in the NTS (Bai et al., 2019; Williams et al., 2016). Consistently, different activity pattern in the NTS following stimulation of either vagal afferent population could be determined (Figures 3D and 3E; Bai et al., 2019). The regulation of adjacent parasympathetic preganglionic neurons in the dorsal motor nucleus of the vagus (DMV) and the control of vagal efferent outflow could relate to the different glucoregulatory functions. Specifically, GABAergic NTS neurons, which send local projections (Babic et al., 2011), constitute the largest population of activated neurons following GLP1R and GPR65 vagal afferent stimulation. Of relevance, it was recently shown that acute stimulation of GABAergic neurons in the NTS inhibits DMV neurons and increases blood glucose levels (Boychuk et al., 2019). Additionally, other downstream brain sites that regulate sympathetic or parasympathetic output could be involved (Steinert et al., 2017). PBd^CCK^, which are selectively activated upon GPR65 vagal afferent stimulation, presumably represent crucial effectors of these specific responses (Flak et al., 2014; Garfield et al., 2014). Future single-cell sequencing studies coupled to circuit mapping and physiological experiments will further determine the genetic identity of the NTS neurons that are downstream of the different vagal afferents.

In summary, we developed an intersectional targeting approach, which is broadly applicable for mapping and manipulating highly selective molecularly defined sensory neurons. This approach allowed the discovery of gut-innervating vagal afferent populations that differently control glucose tolerance and HGP, which is remarkable given that the majority of studies implicates a homogeneous glucoregulatory function of vagal afferents. Given the recent identification of genetically distinct vagal and spinal sensory neurons that innervate different organs of the GI tract (Bai et al., 2019; Hockley et al., 2019), our intersectional approach, coupled with existing or newly generated transgenic mouse lines, provides a mean for future functional interrogation of these neurons in gut-brain communication in normal and disease states.

### Limitations of study

Our study employs an intersectional genetic approach that allows mapping and manipulating Nav1.8-expressing sensory neuron subpopulations. This approach is not limited by the injection of recombinase-dependent viruses and therefore provides a platform for non-invasively controlling transgene expression in individual vagal and spinal afferent populations. However, one limitation of this approach is that a proportion of sensory neurons lacks Nav1.8 and is therefore not intersectionally targeted. This applies, for example, to a subgroup of stomach-innervating vagal afferents as determined through previous genetic mapping and single-cell sequencing analyses (Bai et al., 2019; Gautron et al., 2011). In addition, although PHOX2B vagal afferents and WNT1 sensory neurons intersectionally targeted by the *Nav1.8-p2a-Dre* driver densely innervate GI tract organs, they also innervate other organs and peripheral areas. Thus, it is possible that the observed increases in feeding upon chemogenetic inhibition of these broad populations are due to, at least in part, the decreased activity of non-GI tract innervating sensory neurons. These limitations need to be taken into consideration while interpreting our findings.

## STAR★Methods

### Key resources table

**Table:** 

REAGENT or RESOURCE	SOURCE	IDENTIFIER
**Antibodies**		

Anti-rabbit Alexa594	Invitrogen	Cat#21207; RRID: AB_141637
Rabbit polyclonal anti-dsRed	Living Colors	Cat#632496; RRID: AB_10013483

**Chemicals, peptides, and recombinant proteins**		

20% glucose	DeltaSelect	N/A
2-deoxy-D-[1-14C]-glucose	American Radiolabeled Chemicals	Cat#ARC0111A
40% glucose	bela-pharm	Cat#K4912-03
Cholecystokinin (CCK) Fragment 26-33 Amide (CCK-8)	Sigma-Aldrich	Cat#C2901
Clozapine N-oxide (CNO)	Hello Bio	Cat#HB6149
Clozapine N-oxide (CNO) (Electrophysiology)	Abcam	Cat#141704
Collagenase type 3	Worthington	Cat#LS004182
D-[3-3H]-glucose	PerkinElmer	Cat#NET331A001MC
Insulin (Hyperinsulinemic-euglycemic clamp)	Lilly Deutschland GmbH	HUMINSULIN Normal 100
Insulin (ITT)	Novo Nordisk	Actrapid
Lipopolysaccharide, Salmonella typhimurium (LPS)	Sigma-Aldrich	Cat#C437650
Liraglutide	Novo Nordisk	Victoza
Lithium chloride (LiCl)	Fisher Chemical	Cat#7447-41-8
QIAzol Lysis Reagent	Qiagen	Cat# 79306

**Critical commercial assays**		

Corticosterone Parameter Assay Kit	R&D Systems	Cat#KGE009
Glucagon ELISA	Mercodia	Cat#10-2371-01
High-Capacity cDNA Reverse Transcription Kit	Applied Biosystems	Cat#4368814
RNAscope Multiplex Fluorescent Reagent Kit v2	ACD bio / Bio-Techne	Cat#323100
RNAscope Target Retrieval Reagents	ACD bio / Bio-Techne	Cat#322000
Takyon Low ROX Probe MasterMix	Eurogentec	Cat#UF-LPMT-B0701
TSA PLUS Fluorescence Kits	Perkin-Elmer	Cat#NEL760001KT
Ultra Sensitive Mouse Insulin ELISA Kit	Crystal Chem	Cat#90080

**Experimental models: Organisms/strains**		

Mouse: B6(Cg)-Tg(Phox2b-cre)3Jke/J	The Jackson Laboratory	RRID: IMSR_JAX:016223
Mouse: B6.129-*Trpv1*^*tm1(cre)Bbm*^/J	The Jackson Laboratory	RRID: IMSR_JAX:017769
Mouse: B6.Cg-*H2az2*^*Tg(Wnt1-cre)*11^*R*^*th*^ Tg(Wnt1-GAL4)11Rth/J	The Jackson Laboratory	RRID: IMSR_JAX:009107
Mouse: B6;129S- *Gt(ROSA)26Sor*^*tm66.1(CAG-tdTomato)Hze*^/J	The Jackson Laboratory	RRID: IMSR_JAX:021876
Mouse: B6;129S-*Slc17a8*^*tm1.1(cre)Hze*^/J	The Jackson Laboratory	RRID: IMSR_JAX:028534
Mouse: B6;129S-*Tac1*^*tm1.1(cre)Hze*^/J	The Jackson Laboratory	RRID: IMSR_JAX:021877
Mouse: Nav1.8-p2a-Dre	This paper	N/A
Mouse: R26-LSL-RSR-hM3Dq-ZsGreen	Biglari et al., 2021	N/A
Mouse: R26-LSL-RSR-hM4Di-ZsGreen	This paper	N/A
Mouse: R26-RSR-ZsGreen	Löhr et al., 2018	N/A
Mouse: *Sst*^*tm2.1(cre)Zjh*^/J	The Jackson Laboratory	RRID: IMSR_JAX:013044
Mouse: *Glp1r*^*tm1.1(cre)Lbrl*^/RcngJ	The Jackson Laboratory	RRID: IMSR_JAX:029283
Mouse: *Gpr65*^*tm1.1(cre)Lbrl*^/RcngJ	The Jackson Laboratory	RRID: IMSR_JAX:029282

**Oligonucleotides**		

*G6pc* (Mm00839363_m1)	Eurogentec	N/A
*Hprt* (Mm01545399_m1)	Eurogentec	N/A
*Pck1* (Mm00440636_m1)	Eurogentec	N/A

**Software and algorithms**		

Biorender	Biorender	https://biorender.com/
Clampfit	Molecular Devices	https://www.moleculardevices.com/
Illustrator CC	Adobe Systems	https://www.adobe.com/products/illustrator
ImageJ	Schneider et al., 2012	https://imagej.nih.gov/ij/
pCLAMP 10.7	Molecular Devices	https://www.moleculardevices.com/
Photoshop CC.	Adobe Systems	https://www.adobe.com/Photoshop
Prism	GraphPad	https://www.graphpad.com/scientificsoftware/prism/
SigmaPlot	Systat Software	https://systatsoftware.com
Vinci software package 4.61.0	Cízek et al., 2004	https://vinci.sf.mpg.de/

**Other**		

RNAscope DAPI	ACD bio / Bio-Techne	Cat#320858
RNAscope hydrogen peroxide	ACD bio / Bio-Techne	Cat#322381
RNAscope Protease Plus	ACD bio / Bio-Techne	Cat#322331
smFISH probe: Mm-Calca-tv2tv3-C1 (probe region: 63 – 995 (Accession No. NM_001033954.3))	ACD bio / Bio-Techne	Cat#420361
smFISH probe: Mm-Cckar-C1 (probe region: 328 – 1434 (Accession No. NM_009827.2))	ACD bio / Bio-Techne	Cat#313751
smFISH probe: Mm-Cck-C1 (probe region: 23 – 679 (Accession No. NM_031161.3))	ACD bio / Bio-Techne	Cat#402271
smFISH probe: Mm-Dbh-C1 (probe region: 315 – 1296 (Accession No. NM_138942.3))	ACD bio / Bio-Techne	Cat#407851
smFISH probe: Mm-Fos-C2 (probe region: 407 – 1427 (Accession No. NM_010234.2))	ACD bio / Bio-Techne	Cat#316921-C2
smFISH probe: Mm-Gcg-C1 (probe region: 325 – 939 (Accession No. NM_008100.3))	ACD bio / Bio-Techne	Cat#400601
smFISH probe: Mm-Glp1r-C3 (probe region: 108 – 1203 (Accession No. NM_021332.2))	ACD bio / Bio-Techne	Cat#418851-C3
smFISH probe: Mm-Gpr65-C1 (probe region: 521 – 1652 (Accession No. NM_008152.3))	ACD bio / Bio-Techne	Cat#431431
smFISH probe: Mm-Npy2r-C1 (probe region: 201 – 1059 (Accession No. NM_001205099.1))	ACD bio / Bio-Techne	Cat#315951
smFISH probe: Mm-Npy-C1 (probe region: 28 – 548 (Accession No. NM_023456.2))	ACD bio / Bio-Techne	Cat#313321
smFISH probe: Mm-Phox2b-C3 (probe region: 1617 – 2790 (Accession No. NM_008888.3))	ACD bio / Bio-Techne	Cat#407861-C3
smFISH probe: Mm-Prdm12-C1 (probe region: 64 – 991 (Accession No. NM_001123362.1))	ACD bio / Bio-Techne	Cat#524371
smFISH probe: Mm-Scn10a-C1 (probe region: 2 – 1038 (Accession No. NM_017247.1))	ACD bio / Bio-Techne	Cat#403971
smFISH probe: Mm-Slc17a8-C1 (probe region: 781 – 1695 (Accession No. NM_182959.3))	ACD bio / Bio-Techne	Cat#431261
smFISH probe: Mm-Slc32a1-C3 (probe region: 894 – 2037 (Accession No. NM_009508.2))	ACD bio / Bio-Techne	Cat#319191-C3
smFISH probe: Mm-Sst-C1 (probe region: 18 – 407 (Accession No. NM_009215.1))	ACD bio / Bio-Techne	Cat#404631
smFISH probe: Mm-Tac1-C1 (probe region: 20 – 1034 (Accession No. NM_009311.2))	ACD bio / Bio-Techne	Cat#410351
smFISH probe: Mm-Trpv1-C1 (probe region: 1162 – 2155 (Accession No. NM_001001445.1))	ACD bio / Bio-Techne	Cat#313331
smFISH probe: tdTomato-C2 (probe region: 7 – 1382 (Accession No. N/A))	ACD bio / Bio-Techne	Cat#317041-C2
smFISH probe: ZsGreen-C2 (probe region: 980 – 1655 (Accession No. N/A))	ACD bio / Bio-Techne	Cat#461251-C2

### Resource availability

#### Lead contact

Additional information and requests for resources and reagents should be directed to and will be fulfilled by the Lead Contact, Henning Fenselau (henning.fenselau@sf.mpg.de).

#### Materials availability

Mouse lines generated in this study will be made available upon reasonable request following approval by an internal review board and require a completed Materials Transfer Agreement.

#### Data and code availability

All dataset generated or analyzed during this study are included in the published article. Detailed datasets supporting the current study are available from the Lead Contact upon request. This study did not generate new codes.

### Experimental model and subject details

#### Animals

All experimental procedures were conducted in compliance with protocols approved by local government authorities (Bezirksregierung Köln). Mice were monitored for health status daily, housed at 22–24 ^o^C on a 12 h light/12 h dark cycle, and had ad libitum access to water and to a standard rodent chow diet (ssniff, V1554), unless food was withdrawn for a specific experiment. For all behavioral studies male adult mice were used. For histological and electrophysiological studies adult male and female mice were used.

#### Nav1.8-p2a-Dre mice

*Nav1.8-p2a-Dre* mice were generated using the CRISPR/Cas9 system. A 1337-base ssDNA donor containing the *p2a-Dre* cassette was designed, flanked by 100-base left and right homology arms targeting the *Scn10a* gene just downstream of the stop codon (exon 28). For insertion, two single-guide RNAs (sgRNAs) were engineered to cut the genome close to the homology arms. ssDNA donor, sgRNAs and *Cas9* protein were injected into mouse fertilized eggs from FVB mice using an efficient addition with ssDNA inserts–CRISPR (Easi-CRISPR) genome engineering protocol (Miura et al., 2018). Specific PCR reactions were performed for selecting offspring carrying the correct insertion.

#### R26-LSL-RSR-hM4Di-ZsGreen mice

A Rosa26 locus-targeting vector (B9-36) was designed in which a loxP-flanked STOP cassette and a rox-flanked STOP cassette prevent CAGS promoter-driven expression of the hM4Di and 2A driven ZsGreen. The 5’-primer used for the amplification of *hM4Di* contained an AscI site as well as a Kozak consensus sequence (5Aschm4D: ggcgcgccacc ATGGCCAACTTCACACCTGT) and the 3’-primer contained an AscI site plus one C to keep in frame 2A-ZsGreen translation (3Aschm4Dnew: GGC GCG CCC TGGATCCCGCCTGGCAGT). The sequence-verified *hM4Di* construct was cloned into the AscI-digested B9-36 targeting construct. After vector transfection into Bruce 4 embryonic stem (ES) cells, clonal screening for correct integration was performed by standard Southern blot method. Correctly targeted and verified ES cell clones were chosen for blastocyst injection carried out at Taconic Biosciences to obtain chimeric animals. Resulting chimeras were backcrossed with C57BL/6N animals to obtain germline transmission of the R26-LSL-RSR-hM4Di-ZsGreen allele on a C57BL/6N background.

#### R26-LSL-RSR-hM3Dq-ZsGreen Mice

*R26-LSL-RSR-hM3Dq-ZsGreen* were previously described (Biglari et al., 2021). Briefly, a ROSA26 locus-targeting vector (B9-36) was designed in which both a loxP-flanked STOP cassette and a rox-flanked STOP cassette prevent CAGS promoter-driven expression of the hM3Dq construct. The sequence-verified hM3Dq construct was cloned into the AscI-digested B9-36 targeting construct. Bruce 4 ES cells were used to transfect the vector into and screened for correct integration by Southern blot. Correct ES cell clones were used for blastocyst injection carried out by Taconic Biosciences to obtain chimeric animals. Resulting chimeras were backcrossed with C57BL6 mice to obtain germline transmission on a pure C57BL6 background.

#### Reporter lines

*R26-RSR-ZsGreen* (Löhr et al., 2018), *R26-RSR-LSL-tdTomato* (Madisen et al., 2015) (JAX# 021876) were previously described.

#### Cre lines

*Phox2b-Cre* (Scott et al., 2011) (JAX# 016223), *Wnt1-Cre* (Chai et al., 2000) (JAX# 009107), *Trpv1-ires-Cre* (Cavanaugh et al., 2011) (JAX# 017769), *Tac1-ires-Cre* (Harris et al., 2014) (JAX# 021877), *Sst-ires-Cre* (Taniguchi et al., 2011) (JAX# 013044), *Vglut3-ires-Cre* (Tasic et al., 2018) (JAX# 028534), *Glp1r*-ires-*Cre* (Williams et al., 2016) (JAX# 029283), *Gpr65-ires-Cre* (Williams et al., 2016) (JAX# 029282) were previously described and purchased from Jackson Laboratories.

#### Breeding scheme and genetic backgrounds

All transgenic animals were bred to C57BL6 mice for maintenance. Triple transgenic animals and control mice were generated by crossing Cre mice with double transgenic mice (*Nav1.8-p2a-Dre*; *R26-LSL-RSR-hM3Dq-ZsGreen or – hM4Di-ZsGreen*, or *R26-RSR-LSL-tdTomato*) from a mixed genetic background (129/C57BL6). Control animals were littermates to the experimental triple transgenic mice and were of either single transgenic or double transgenic (any of the possible combinations), or nontransgenic genotypes.

### Method details

#### Organ tissue preparation

For immunostaining and in situ hybridization studies, mice were deeply anesthetized and transcardially perfused with PBS followed by 4% paraformaldehyde (PFA) in PBS (PFA-PBS). Organs were dissected, post-fixed at 4°C in PFA-PBS for variable periods of time (brains and spinal cords for 6 hours, NG and DRG for 24 hours, other organs for 48 hours,) and then transferred to 20% sucrose in PBS. Brainstems were cut in 30 μm sections for immunostaining and 18 μm sections for FISH using a microtome, and every fourth section was further processed for immunohistochemistry or FISH as described below. Spinal cords, NG, DRG, and other organs were cut using a cryostat. Spinal cords were coronally cut in 18 μm thick sections. Stomachs were subdivided in antrum, corpus and fundus, and intestines in duodenum (0-3 cm from pylorus), jejunum (6-9 cm from pylorus), ileum (6 cm from cecum) and colon (0-2 cm after cecum), and cut in 18 μm transverse thick sections. NG and DRG were cut in 14 μm thick sections. Trachea, hearts, lungs, livers, gallbladders and kidneys were cut in 18 μm transverse thick sections.

#### Immunohistochemistry

Sections were blocked with 2% normal donkey serum in 0,4% Triton X-100 in PBS (NDS-PBST) for 1 hour at room temperature (RT) and incubated with anti-dsRed antibody (1:1000, rabbit, Living Colors #632496; RRID:AB_10013483) diluted in NDS-PBST overnight at RT. Sections were washed with PBST and then incubated with a secondary antibody anti-rabbit Alexa594 (1:1000, donkey, Invitrogen #A21207; RRID:AB_141637) diluted in PBS for 1 hour at RT. After several washes with PBS, sections were counterstained with DAPI containing mounting medium (VECTASHIELD Antifade Mounting Medium with DAPI, Cat# H-1200, Vector Laboratories), mounted and imaged by a Zeiss ImagerM2 fluorescent microscope with 10x or 20x magnification, or a Leica TCA SP-8-X Confocal Microscope (Leica Microsystems) with 20x magnification.

#### *In situ* hybridization

RNAscope Multiplex Fluorescent Reagent Kit v2 (Advanced Cell Diagnostic, Cat# 323100) was used following the manufactures' instructions. Sections were dried at 60°C overnight, pre-treated with hydrogen peroxide (Cat# 322381), and boiled in Target retrieval (Cat# 322000). After dehydrating in pure ethanol, sections were surrounded by a hydrophobic barrier (ImmEdge hydrophobic barrier pen, Vector Lab, H-4000) and incubated in Protease Plus (Cat# 322331; 15 min at 40°C) followed by the target probes (Mm-Scn10a-C1 (Nav1.8), Cat# 403971; tdTomato-C2, Cat# 317041-C2; ZsGreen-C2, Cat# 461251-C2; Mm-Phox2b-C3, Cat# 407861-C3; Mm-Prdm12-C1, Cat# 524371; Mm-Trpv1-C1, Cat# 313331; Mm-Tac1-C1, Cat# 410351; Mm-Sst-C1, Cat# 404631; Mm-Slc17a8-C1 (Vglut3), Cat# 431261; Mm-GLP1R-C3, Cat# 418851-C3; Mm-GPR65-C1, Cat# 431431; Mm-Fos-C2, Cat# 316921-C2; Mm-Calca-tv2tv3-C1, Cat #420361; Mm-Npy2r-C1, Cat# 315951; Mm-Cckar-C1, Cat#313751; Mm-Npy-C1, Cat# 313321; Mm-Gcg-C1, Cat# 400601; Mm-Slc32a1-C3, Cat# 319191-C3, Mm-Cck-C1, Cat# 402271; Mm-Dbh-C1, Cat# 407851; 2 hours at 40°C) in a HybEZ oven. Signal amplification was reached using amplifiers AMP1-3 and label probes (Opal520, Cy3 and Cy5; Perkin-Elmer, Cat# NEL760001KT). Sections were mounted using DAPI containing mounting medium (VECTASHIELD, Cat# H-1200, Vector Laboratories). Slides were imaged by a Zeiss ImagerM2 fluorescent microscope with 10x or 20x magnification or Leica TCA SP-8-X Confocal Microscope (Leica Microsystems) with 20x magnification.

#### Analysis of stained tissues

Images were processed using ImageJ software (Schneider et al., 2012) and stitched together using the plugin Stitching (Preibisch et al., 2009). Background was subtracted and images were cropped. Neurons positive for RNAscope probes in NG and DRG were quantified manually using ImageJ software from 3-4 different ganglia within a fixed area (340x340 pixels), positioned randomly. For analysis of terminal endings in muscular and mucosal layers, representative images of GI tract organs were taken. Muscular and mucosal layers were identified by their autofluorescence in the 590 channel, and the number of tdTomato-containing terminal endings within these layers was analyzed. Terminal endings were quantified manually using ImageJ software from 3 different slices (stomach: 3500x3500 pixels; small and large intestine: 2000x2000 pixels). Innervation ratio of villi was quantified manually using ImageJ software. The total number of villi and the number of villi that contained at least one terminal were assessed. tdTomato-containing endings in other abdominal organs were analyzed manually.

#### Fos analysis

Mice were injected with 3 mg/kg Clozapine-N-Oxid (Cat# HB6149, CNO dihydrochloride (water soluble), Hello Bio, Dunslaughlin, IE) in saline i.p.. 45 minutes later animals were deeply anesthetized and transcardially perfused with PBS followed by 4% paraformaldehyde (PFA) in PBS (PFA-PBS). Brains were dissected, post-fixed at 4°C in PFA-PBS for 12 hours and then transferred to 20% sucrose in PBS. Brainstems were cut in 16 μm sections after a minimum time of 12h in sucrose solution using a microtome, and every fourth section was further processed for FISH. Images were processed using ImageJ software (NIH). Background was subtracted and images were cropped. For Fos analysis in NTS and AP, one section of each Bregma (-7.20, -7.48, and -7.92mm) was chosen from each animal (n=2-3 per group). Anatomical landmarks (AP, central canal) were determined according to a mouse brain atlas (https://mouse.brain-map.org/static/atlas) and cells positive for *Fos* mRNA were counted. For Fos analysis in the PB, 3 sections containing the PB (Bregma -5.02 to -5.20mm) were chosen and analyzed; for the PBe and the PBd separately. Anatomical landmarks were determined as the superior cerebellar peduncle, ventral spinocerebellar tract, and the cerebral aquaeduct. For Fos analysis following LiCl injection, 84 mg/kg LiCl (Cat# 7447-41-8, Fisher Chemical, Thermo Fisher Scientific, MA, USA) in saline was injected i.p. 45 minutes after CNO injection, and mice were sacrificed 45 minutes later. Tissue was processed as described above.

#### Colocalization analysis

Expression of *Fos* and one molecular marker (*Dbh, Npy, Cck, Gcg* or Vgat (*Slc32a1*)) in the NTS, or *Calca* and *Cck* in the PB, were determined using FISH. Co-expressing cells were counted in the NTS, PBe, or PBd. Colocalization is reported as total number of Fos expressing *Dbh, Npy, Cck, Gcg, Calca*, or Vgat cells per analyzed section, or percentage of Fos+ Calca+ neurons for LiCl experiments.

#### Electrophysiology

Whole cell patch clamp recordings from sensory neurons were performed on entire ganglia as previously described (Ciglieri et al., 2016). Mice were deeply anesthetized, decapitated, DRG were removed, and immediately placed into ice-cold solution, containing (in mM): sucrose 252, KCl 2.5, NaHCO_3_ 26, NaH2PO4 1.25, D-glucose 10, kynurenate 1, MgCl2 3, CaCl2 1.5, oxygenated with 95% O_2_/5% CO_2_. DRG were moved into artificial cerebrospinal fluid (aCSF; as below) containing collagenase (7 mg/mL, collagenase type 3; Worthington, NJ, USA) and incubated for 1 h at 35°C. A single ganglion was transferred into a recording chamber, where it was constantly superfused with aCSF (2 mL/min), containing (in mM): NaCl 126, KCl 2.5, D-glucose 10, NaHCO_3_ 26, NaH2PO4 1.25, CaCl2 2, MgCl2 1.5, oxygenated with 95% O_2_/5% CO_2_. Recordings were performed at room temperature using glass electrodes with ∼5 MΩ resistance filled with an internal solution containing (in mM): KMeSO3 135, HEPES 10, EGTA 1, MgCl2 4, Na2ATP 4, Na2GTP 0.4, Na2-Phosphocreatine 5, CaCl2 0.1, and sucrose 5, pH adjusted to 7.3 (with KOH). Neurons were visualized using an upright microscope (SliceScope; Scientifica, Uckfield, UK) equipped with a 40x water immersion objective (Olympus, Tokyo, Japan) and a CCD camera (SciCam Pro; Scientifica, Uckfield, UK). Recordings were performed using a Multiclamp 700B amplifier (Molecular Devices, Sunnyvale, USA) connected to a Digidata interface (Digidata 1550B; Axon Instruments, Union City, USA), sampled at 10 kHz and filtered at 2 kHz. Signals were recorded using pCLAMP 10.7 (Molecular Devices, Sunnyvale, USA).

Recordings were included for analysis when access resistance changed less than 20% throughout the recordings. Neurons were recorded in voltage-clamp mode before and after CNO (abcam, Cat# ab141704) administration to assess inhibitory currents elicited by two protocols: 1) A ramp-and-hold protocol in current-clamp mode consisting of a 250 ms ramp followed by 500 ms of continuous current injection. 2) A depolarizing protocol in voltage clamp mode in which each neuron was kept at a holding potential of -80 mV and depolarized to -40 mV in 5 mV incrementing steps of 100ms. Analysis was performed offline using Clampfit (Molecular Devices). To normalize for cell variations, current amplitudes were normalized (I_max_/I). I-V curves of the depolarizing protocol were obtained by measuring the current amplitude at the peak present at the beginning of each voltage step.

#### Food intake studies

All animals were singly housed and handled for at least 7 consecutive days before the assay to acclimate mice to the experimental procedure. Feeding studies were performed in home cages with ad libitum food access to chow. Before the experiment, mice were provided with fresh cages to avoid leftover food spilling in the bedding. CNO was diluted in saline and administered at 1-3 mg per kg of body weight.

For light-cycle measurements, animals were injected i.p. with CNO 3 hours after onset of the light cycle, and food intake was monitored hourly for 4 hours after injection. For refeeding experiments, mice were provided with fresh cages one hour before onset of the dark cycle on the day before the experiment and no food was provided. After 16 hours fasting, mice were i.p. injected with CNO 3 hours after onset of the light cycle and food intake was monitored hourly for 4 hours after injection. For analysis of the effect of anorexigenic agents, LiCl (84 mg/kg), LPS (25 μg/kg, Cat# 437650, Sigma Aldrich, MO, USA), CCK-8 (40 μg/kg, Cat# C2901, Sigma Aldrich, MO, USA) or Liraglutide (25 μg/kg, or 200 μg/kg respectively, Victoza, Novo Nordisk, Bagsvaerd, Denmark) diluted in saline were injected 15 minutes before refeeding (Figure 4E). For dark-cycle measurements, animals were i.p. injected with CNO one hour prior to the onset of the dark cycle and food intake was monitored hourly for 4 hours after injection.

#### Serology

Control and triple transgenic mice serum was collected from small tail incisions or from trunk blood either 1 hour after i.p. injection of CNO or at the end of the euglycemic-hyperinsulinemic clamp studies. Levels of insulin (Cat# 90080, Crystal Chem), glucagon (Cat# 10-2371-01, Mercodia), or corticosterone (Cat# KGE009, R&D Systems) were determined using commercially available enzyme-linked immunosorbent assays according to the manufacturer's instructions.

#### Glucose-tolerance tests

GTTs were performed after a fasting period of 16 hours. Blood glucose concentrations were measured from whole venous blood using an automatic glucose monitor (Bayer HealthCare Ascensia Contour). Each mouse received an intraperitoneal injection of 20% glucose (10 ml per kg body weight; DeltaSelect) and blood glucose concentrations were measured at baseline and after 15, 30, 60 and 120 min. Chemogenetic activation of hM3Dq-expressing sensory neurons was achieved by injecting CNO one hour prior to glucose administration.

#### Glucose tolerance tests after i.p. injection of agents

GTTs were performed as described above. For analysis of blood glucose modulation by different agents, liraglutide (25 μg/kg, or 200 μg/kg respectively) or CCK-8 (40μg/kg) were injected i.p. 15 minutes before glucose injection.

#### Insulin-tolerance tests

ITTs were performed at the beginning of the light cycle in fed mice. After determination of basal blood glucose concentrations, each mouse received an intraperitoneal injection of insulin (0.375 (chemogenetic activation studies) or 0.75 (chemogenetic inhibition studies) iU per kg body weight, Actrapid, Novo Nordisk) and glucose concentrations in blood were measured after 15, 30, 60and 120 min. Chemogenetic activation of hM3Dq-expressing or inhibition of hM4Di expressing sensory neurons, respectively, was achieved by injecting CNO one hour prior to insulin administration. Food was removed from the cages after CNO administration during ITTs.

#### Blood glucose measurements during dark cycle feeding

Blood glucose concentrations were measured during dark cycle feeding. CNO was administrated one hour prior to the beginning of the dark cycle and blood glucose was assessed at baseline and hourly for 4 hours from whole venous blood using an automatic glucose monitor (Bayer HealthCare Ascensia Contour).

#### Euglycemic-Hyperinsulinemic clamp studies in awake mice

Catheters were implanted into the jugular vein and clamp procedures were performed as previously described (Steculorum et al., 2016). Briefly, 5–6 days post catheter implantation, mice with less than 15% reduction from their preoperative body weight were subjected to the clamp studies. On the day of the experiment, food was removed from cages 2 hours after onset of the light cycle. Four hours later, mice were injected with CNO and placed in a customized cage for the clamp procedures. D-[3-^3^H]-glucose (PerkinElmer, Cat# NET331A001MC) was administered as a bolus (0.8 μCi) followed by continuous administration of D-[3-^3^H]-glucose (0.04 Ci/min) in 3% plasma solution (from C57BL/6 mice). Hyperinsulinemia was induced by continuous infusion of insulin (4 μU/g/min; HUMINSULIN Normal 100, Lilly Deutschland GmbH). CNO was added to the insulin solution to deliver a total of 2 mg per kg bodyweight until the end of the clamp (0.0125 mg/kg/min). Glycemia was monitored regularly from tail vein bleeding (Hemocue Glucose 201 RT) and kept at 140 mg/dL through the infusion of 40% glucose (bela-pharm, Cat# K4912-03) together with D-[3-^3^H]-glucose (0.04 μCi/μL). Steady state was considered to be achieved when a fixed glucose infusion rate was able to maintain constant glycemia for at least 30 min. 2-deoxy-D-[1-^14^C]-glucose (10 μCi; American Radiolabeled Chemicals, Cat# ARC0111A) was administered for analysis of tissue specific uptake. Mice were sacrificed through decapitation and trunk blood, gonadal white adipose tissue, brown adipose tissue, livers and skeletal muscle were collected, and stored at -80°C until further analysis. Plasma [3-3H]-glucose content at basal and steady state was assessed. Collected tissues were lysed and processed through ion-exchange chromatography columns (Poly-Prep Prefilled; Bio Rad, Cat# 731-6221) to measure the accumulation of 2-deoxy-D-[1-14C]-glucose and its disappearance from serum. All studies were performed using a liquid scintillation analyser (PerkinElmer, Tri-Carb 2810TR). All the data obtained were analyzed using Prism 8.0 (GraphPad) software and SigmaPlot (Systat Software).

#### Gene expression analysis

Gene expression was assessed on livers collected after euglycemic-hyperinsulinemic clamp procedures. mRNA was isolated using a standard QIAzol/chloroform based protocol (QIAzol Lysis Reagent, Cat# 79306, Qiagen) and reversely transcribed using an High-Capacity cDNA Reverse Transcription Kit (Cat# 4368814, Applied Biosystems). Real-time PCR was performed using QuantStudio7 Flex (Applied Biosystems, Thermo Fisher Scientific) on a mix containing cDNA, Takyon Low ROX Probe MasterMix (Cat# UF-LPMT-B0701, Eurogentec) and the Taqman probe of interest. The probes used were *G6pc* (Mm00839363_m1) and *Pck1* (Mm00440636_m1), whose relative expression was adjusted for the total RNA content by *Hprt* (Mm01545399_m1). The final values of gene expression fold change were calculated using the comparative 2ˆ-ΔΔCT method.

#### Positron emission tomography scans

PET imaging was performed using an Inveon preclinical PET/CT system (Siemens). Mice were injected i.p. with CNO or saline. One hour later, mice were anesthetized with 2% isoflurane in 65%/35% nitrous oxide/oxygen gas and positioned on a dedicated mouse carrier (MEDRES, Germany) carrying two mice. Body temperature was maintained at 37.0±0.5°C by a thermostatically controlled water heating system. For injection of the radiotracer, a catheter consisting of a 30G cannula connected to a polythene tubing (ID=0.28 mm) was inserted into the tail vein and fixated by a drop of glue. Per mouse, 7-8 MBq of [18F]FDG in 50-100 μL saline were injected via tail vein after starting the PET scan. Emission data were acquired for 45 minutes. Thereafter, animals were automatically moved into the CT gantry and a CT scan was performed (180 projections/360°, 200 ms, 80 kV, 500 μA). The CT data were used for attenuation correction of the PET data and the CT image of the skull was used for image co-registration. Plasma glucose levels were determined from a tail vein blood sample using a standard glucometer (Bayer Contour Next, Bayer Vital GmbH) after removing the tail vein catheters. PET data were histogrammed in time frames of 12 x 30s, 3 x 60s, 3 x 120s and 7 x 240s, Fourier rebinned and then images were reconstructed using the MAP-SP algorithm provided by the manufacturer. For co-registration, the imaging analysis software Vinci (Cízek et al., 2004) was used. Images were co-registered to a 3D mouse brain atlas constructed from the 2D mouse brain atlas.

#### Kinetic modeling

An image-derived input function was extracted from the PET data of the aorta, which could be identified in the image of the first time frame of each animal. Input function data were corrected for partial volume effect by assuming a standardized volume fraction of 0.6. Parametric images of the [18F]FDG kinetic constants K1, k2, k3, and k4 were determined by a voxel-by-voxel (voxel size= 0.4 mm x 0.4 mm x 0.8 mm) fitting of data to a two-tissue-compartment kinetic model. K1 is the constant for transport from blood to tissue, k2 for transport from tissue to blood, k3 the constant for phosphorylation of [18F]FDG to [18F]FDG-6-phosphate, and k4 the constant for dephosphorylation. In order to detect variations in glucose metabolism we here use the ratio of tissue to blood glucose (CE/CP) instead of the direct metabolic rate. CE/CP can be calculated from the rate constants as CE/CP=K1/(k2+k3/0.26), is a measure for glucose transport, and – in contrast to the metabolic rate of glucose itself – it is insensitive to changes in the blood glucose level. Since neuronal activation is accompanied by increased glucose transport, alterations of CE/CP can be used as surrogate for alterations in neuronal activation. Statistical testing was performed for each image voxel by application of a t-test between the difference of CNO and saline in triple transgenic mice and control littermates. For presentation only, 3D maps of p-values were re-calculated on a 0.1 mm x 0.1 mm x 0.1 mm grid from the original dataset using trilinear interpolation.

### Quantification and statistical analysis

Statistical analyses were performed using Prism 8.0 (GraphPad) software, unless indicated otherwise. Statistical tests applied are found in the figure legends. No statistical method was used to predetermine sample size. Sample sizes were chosen to be similar to those reported in previous publications (Steculorum et al., 2016) and are reported in the figure legends. Randomization and blinding methods were not used. All data presented met the assumptions of the statistical test employed. Data are presented as mean ± SEM unless indicated otherwise. Boxplots show median (line), quartiles (boxes) and range (whiskers). Statistical significance is represented by ^∗^p ≤ 0.05, ^∗∗^p ≤ 0.01, ^∗∗∗^p ≤ 0.001 and ^∗∗∗∗^p ≤ 0.0001.

## Supplemental information

Document S1. Figures S1–S6 and Table S1

Document S2. Article plus supplemental information
