# Supplementary material for: Gut-brain communication by distinct sensory neurons differently controls feeding and glucose metabolism
Source: Cell Metab. 2021 Jul 6;33(7):1466–1482.e7. doi: 10.1016/j.cmet.2021.05.002 (PMC8280952; doi:10.1016/j.cmet.2021.05.002)
Supplement: Document S1. Figures S1–S6 and Table S1 [file mmc1.pdf]

Cell Metabolism, Volume *33*

Supplemental information

Gut-brain communication by distinct sensory neurons differently controls feeding and glucose metabolism

Diba Borgmann, Elisa Ciglieri, Nasim Biglari, Claus Brandt, Anna Lena Cremer, Heiko Backes, Marc Tittgemeyer, F. Thomas Wunderlich, Jens C. Brüning, and Henning Fenselau

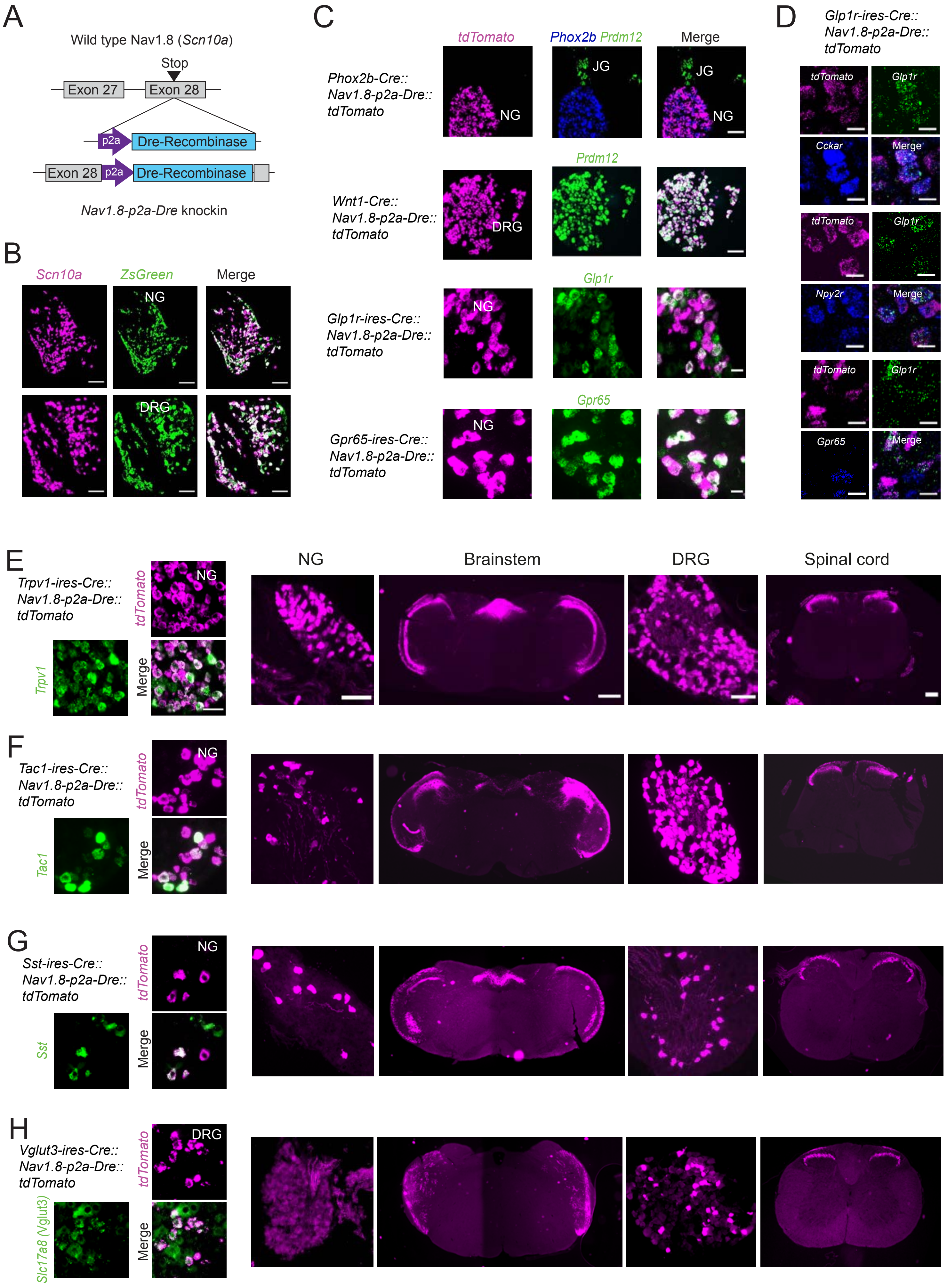

A
Wild type Nav1.8 (Scn10a)
Stop
Exon 27
Exon 28
p2a
Dre-Recombinase
Exon 28
p2a
Dre-Recombinase
Nav1.8-p2a-Dre knockin
B
Scn10a
ZsGreen
Merge
NG
DRG
C
tdTomato
Phox2b Prdm12
Merge
Phox2b-Cre::
Nav1.8-p2a-Dre::
tdTomato
JG
NG
Wnt1-Cre::
Nav1.8-p2a-Dre::
tdTomato
Prdm12
DRG
Glp1r-ires-Cre::
Nav1.8-p2a-Dre::
tdTomato
Glp1r
NG
Gpr65-ires-Cre::
Nav1.8-p2a-Dre::
tdTomato
Gpr65
NG
D
Glp1r-ires-Cre::
Nav1.8-p2a-Dre::
tdTomato
tdTomato
Glp1r
Cckar
Merge
tdTomato
Glp1r
Npy2r
Merge
tdTomato
Glp1r
Gpr65
Merge
E
NG
Brainstem
DRG
Spinal cord
Trpv1-ires-Cre::
Nav1.8-p2a-Dre::
tdTomato
tdTomato
Trpv1
Merge
NG
F
Tac1-ires-Cre::
Nav1.8-p2a-Dre::
tdTomato
tdTomato
Tac1
Merge
NG
G
Sst-ires-Cre::
Nav1.8-p2a-Dre::
tdTomato
tdTomato
Sst
Merge
NG
H
Vglut3-ires-Cre::
Nav1.8-p2a-Dre::
tdTomato
tdTomato
Slc17a8 (Vglut3)
Merge
DRG

Figure S1, Related to Figure 1: Generation and validation of *Nav1.8-p2a-Dre* mice, and intersectional mapping sensory neuron subtypes.

(A) Schematic diagram of the *Nav1.8-p2a-Dre* allele. The p2a-Dre-cassette was targeted to the stop codon of the Nav1.8 (*Scn10a*) gene.

(B) *Nav1.8-p2a-Dre* mice were crossed with Dre-dependent reporter mice for the expression of ZsGreen (Lohr et al., 2018). Colocalization of ZsGreen and endogenous Nav1.8 (*Scn10a*; magenta) mRNA in *Nav1.8-p2a-Dre::ZsGreen* mice assessed by FISH. Scale bars represent 100 µm.

(C) Representative images showing *tdTomato* (magenta) and endogenous mRNA expression in NG and DRG: *Phox2b* (blue), and *Prdm12* (green) in mice derived from *Phox2b-Cre* mice or *Wnt1-Cre* mice; *Glp1r* (green) in mice from *Glp1r-ires-Cre* mice; *Gpr65* (green) in mice from *Gpr65-ires-Cre* mice. No *tdTomato* was observed in DRG in mice derived from *Phox2b-Cre*, *Glp1r-ires-Cre* and *Gpr65-ires-Cre* mice. Scale bars represent 100 µm (*Phox2b-Cre* and *Wnt1-Cre*) and 25 µm (*Glp1r-ires-Cre* and *Gpr65-ires-Cre*).

(D) Representative images showing *tdTomato* (magenta), and endogenous *Glp1r* (green) and *Cckar*, *Npy2r,* or *Gpr65* (blue) expression in NG in mice derived from *Glp1r-ires-Cre* mice. Scale bars represent 20 µm.

(E-H) Left: representative images showing *tdTomato* (magenta) and endogenous *Trpv1* **(E)**, *Tac1* **(F)**, *Sst* **(G)** and *Slc17a8* (Vglut3; green*)* **(H)** in NG or DRG assessed by FISH. Right: tdTomato (magenta) expression in NG, brainstem, DRG, and spinal cord in triple transgenic mice derived from *Trpv1-ires-Cre* **(E)**, *Tac1-ires-Cre* **(F)**, *Sst-ires-Cre* **(G)**, and *Vglut3-ires-Cre* **(H)** mice, assessed by immunohistochemistry.

Scale bars represent 50 µm (FISH, NG), 100 µm (NG and DRG), 500 µm (brainstem), and 200 µm (spinal cord).

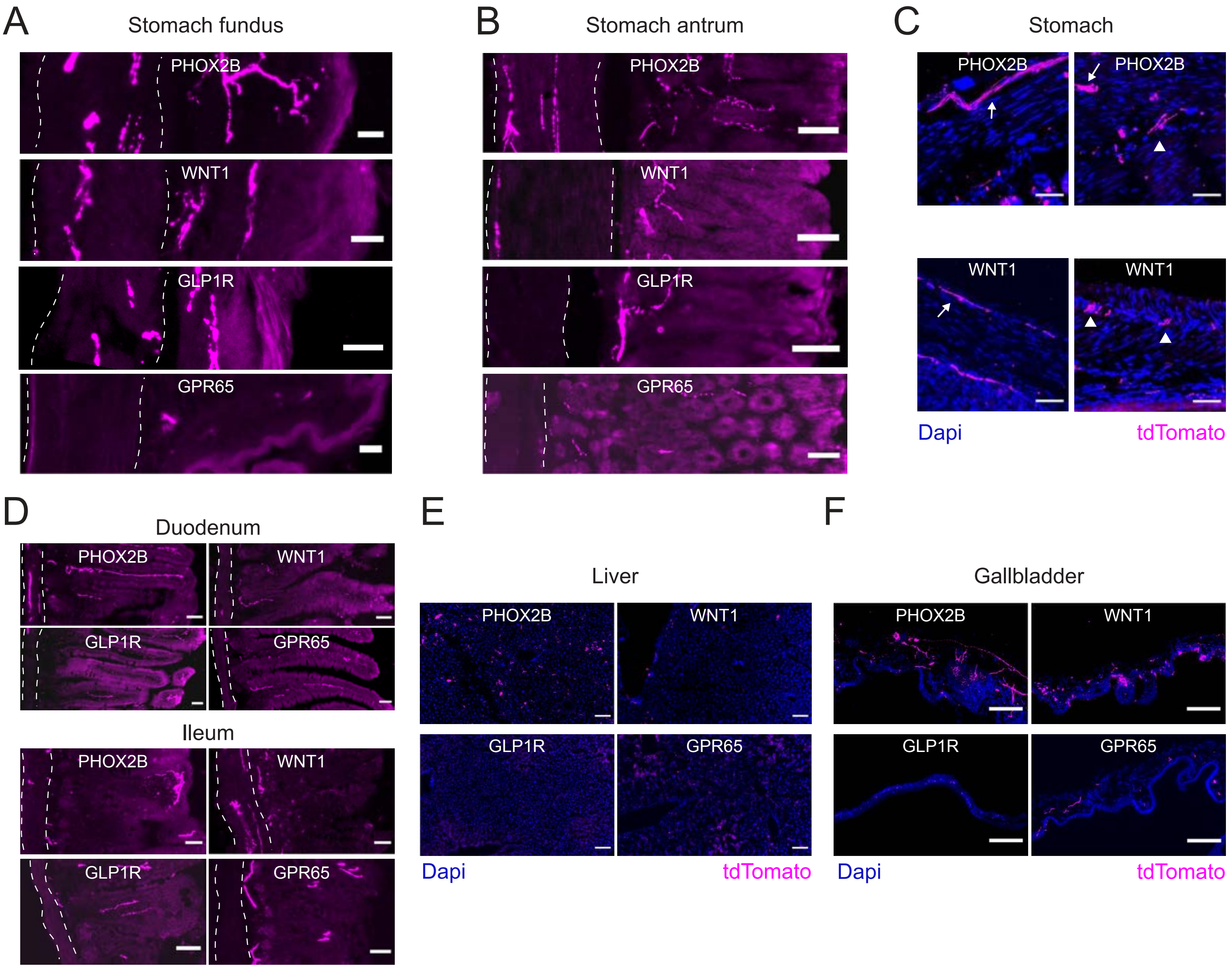

G

Relative innervation

	Trachea	Heart	Lung	Liver	Gallbladder	Kidney
PHOX2B	+	(+)	+	+++	+++	-
WNT1	++++	++	(+)	-	-	(+)
GLP1R	(+)	-	-	-	-	-
GPR65	-	-	-	-	(+)	-

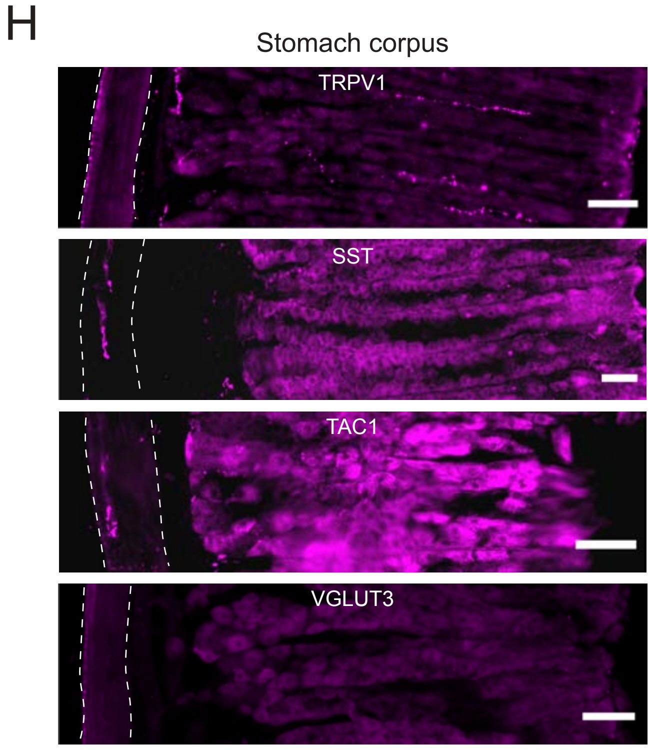

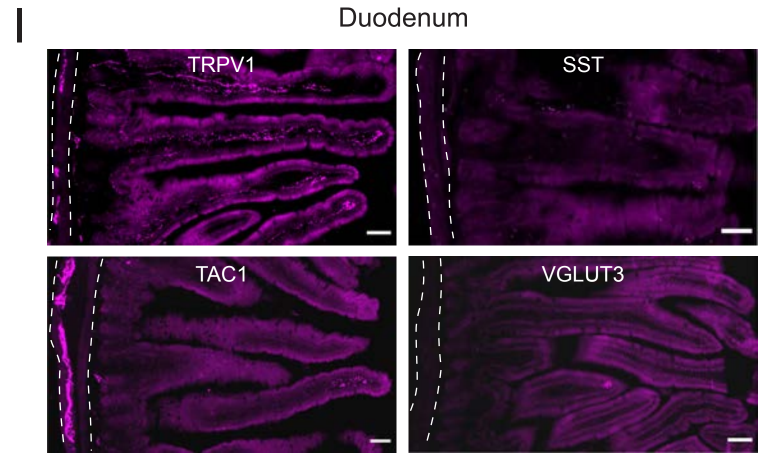

Figure S2, Related to Figure 2: Organ innervation pattern of molecularly-defined sensory neuron populations.

(A-F) Representative images showing tdTomato (magenta) expression in triple transgenic mice derived from *Phox2b-Cre*, *Wnt1-Cre*, *Glp1r-ires-Cre* and *Gpr65-ires-Cre* mice in stomach fundus **(A)** and antrum **(B)**, duodenum and ileum **(D)**, liver **(E)**, and gallbladder **(F)**, assessed by immunohistochemistry.

(C) Representative images showing innervation in muscularis propria of the stomach. Arrows indicate intramuscular array (IMA); arrow heads indicate intraganglionic laminar endings (IGLEs).

(G) Analysis of abdominal organ innervation by sensory neurons in triple transgenic mice derived from *Phox2b-Cre*, *Wnt1-Cre*, *Glp1r-ires-Cre* and *Gpr65-ires-Cre* mice assessed by tdTomato expression.

(H, I) Representative images showing tdTomato (magenta) expression in mice derived from *Trpv1-ires-Cre*, *Sst-ires-Cre*, *Tac1-ires-Cre* and *Vglut3-ires-Cre* mice in stomach corpus **(H)** and duodenum **(I)**.

Scale bars represent 50 µm; 25 µm (fundus) and 100 µm (liver and gallbladder). Dashed lines indicate muscular layer.

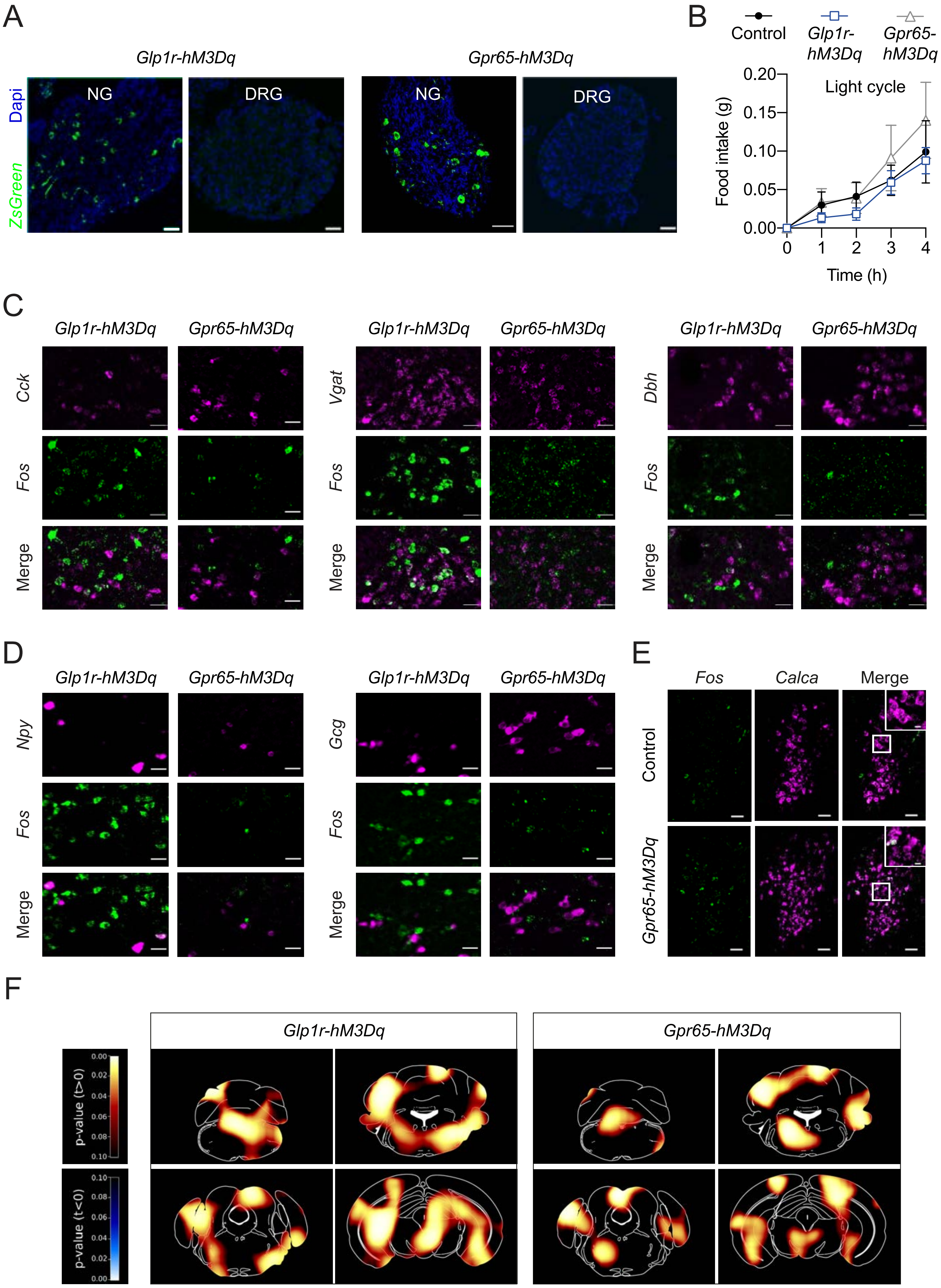

A
Glp1r-hM3Dq
Gpr65-hM3Dq
Dapi
ZsGreen
NG
DRG
B
Control
Glp1r-hM3Dq
Gpr65-hM3Dq
Light cycle
Food intake (g)
0.20
0.15
0.10
0.05
0.00
0
1
2
3
4
Time (h)
C
Cck
Vgat
Dbh
Fos
Merge
D
Npy
Gcg
E
Fos
Calca
Merge
Control
Gpr65-hM3Dq
F
p-value (t>0)
p-value (t<0)
0.00
0.02
0.04
0.06
0.08
0.10

Figure S3, Related to Figure 3: Regulation of feeding neurocircuits by GLP1R and GPR65 vagal afferents.

(A) Expression of hM3Dq-zsGreen in NG and DRG. Scale bars represent 50 µm. Quantitative analysis revealed that the number of GLP1R ZsGreen NG cells was about 2.5-fold as compared to GPR65 ZsGreen NG cells (GLP1R 24.7±4.73 versus GPR65 10±1.87; cells per section).

(B) Light cycle feeding during stimulation of GLP1R and GPR65 vagal afferents. Mice per group n=9-12.

(C-E) Representative images showing *Cck*, *Vgat*, *Dbh*, *Npy*, *Gcg* and *Fos* expression in the NTS (Bregma -7.48 - -7.92) after CNO-induced activation of GLP1R or GPR65 vagal afferents as assessed by FISH. Note that higher *Fos* mRNA was detected in NTS cells in response to GLP1R than GPR65 vagal afferent stimulation.

(F) Brain activation pattern upon stimulation of the two subtypes as assessed by [^{18}F]FDG PET (p values from voxelwise t-tests are indicated by color bar). Mice per group n=9-12

In all experiments, triple transgenic mice and littermate controls were injected with CNO. Mice are from multiple litters. Statistical significance was assessed by two-way mixed effects ANOVA with Dunnett's test for multiple comparisons **(B)** or two-tailed unpaired Student's t-test **(F)**. Values are presented as mean +/- SEM.

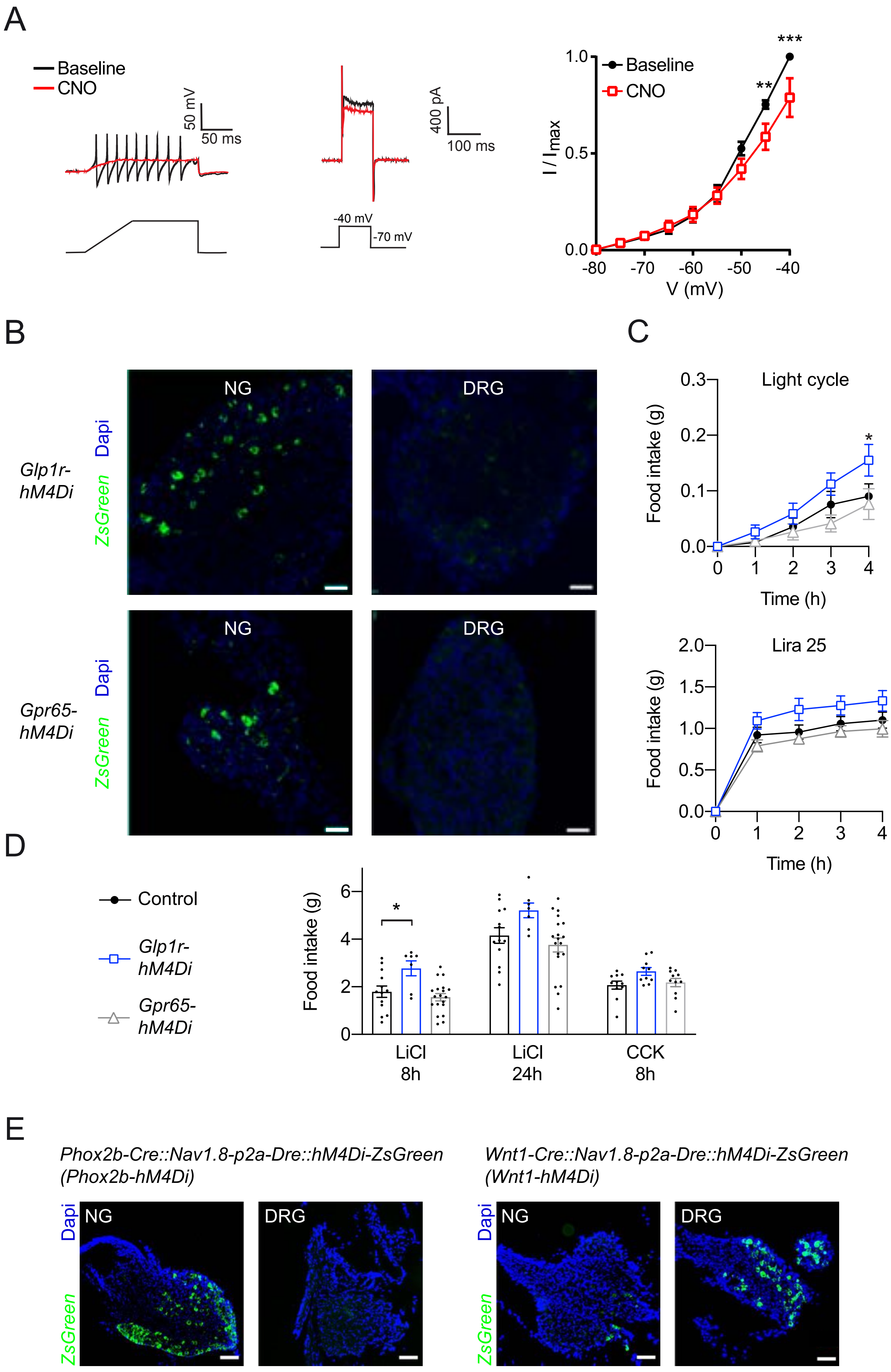
A
Baseline
CNO
50 mV
50 ms
400 pA
100 ms
-40 mV
-70 mV
I / Imax
V (mV)
***
**
B
C
NG
DRG
Glp1r-hM4Di
Gpr65-hM4Di
Dapi
ZsGreen
Light cycle
Lira 25
Food intake (g)
Time (h)
D
Control
Glp1r-hM4Di
Gpr65-hM4Di
LiCl 8h
LiCl 24h
CCK 8h
E
Phox2b-Cre::Nav1.8-p2a-Dre::hM4Di-ZsGreen (Phox2b-hM4Di)
Wnt1-Cre::Nav1.8-p2a-Dre::hM4Di-ZsGreen (Wnt1-hM4Di)

Figure S4, Related to Figure 4: Validation of hM4Di-Zsgreen mice, and alterations in food intake upon inhibition of sensory neuron populations.

(A) Representative traces (left) and summary (right) of CNO effects on hM4Di-expressing neurons. Recordings were performed from ZsGreen-expressing DRG neurons from *Wnt1-Cre-hM4Di* mice. Neurons n=5 (mice n=5).

(B) Expression of hM4Di-ZsGreen in NG and DRG assessed by FISH. Scale bars represent 50 μm.

(C) Effects of chemogenetically inhibiting GLP1R or GPR65 vagal afferents on light cycle feeding (top), and refeeding after 25 μg/kg Liraglutide (bottom). Mice per group n=5-8.

(D) Long-term effects of chemogenetic inhibition of GLP1R or GPR65 vagal afferents on feeding after injection of LiCl or CCK. Mice per group n=7-19.

(E) Representative images showing expression of hM4Di-zsGreen in NG and DRG assessed by FISH. Scale bars represent 100 μm.

In food intake experiments, triple transgenic mice and littermate controls were injected with CNO. Mice are from multiple litters. Statistical significance was assessed by repeated measures ANOVA with Bonferroni correction **(A)**, two-way mixed effects ANOVA **(C)** or ordinary one-way ANOVA **(D)** with Dunnett's test for multiple comparisons.

Signifi-cant results are indicated by * p ≤0.05, ** p≤0.01 and *** p≤0.001. Values are presented as mean +/- SEM.

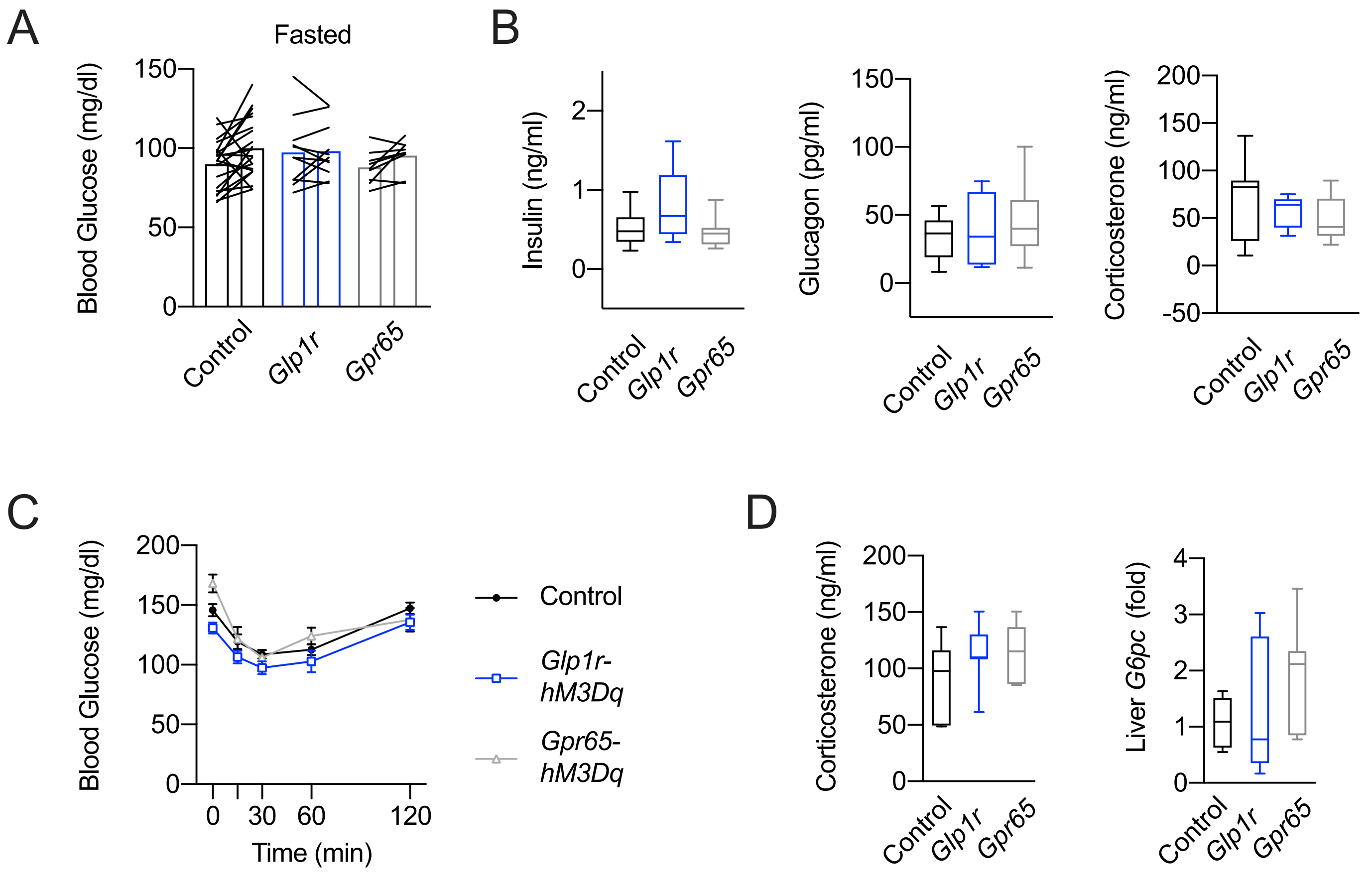

Figure S5, Related to Figure 5: Regulation of glucose metabolism by GLP1R and GPR65 vagal afferents.

(A) Effects of hM3Dq-induced stimulation of GLP1R or GPR65 vagal afferents on blood glucose levels in fasted mice.

(B) Analysis of plasma levels of insulin, glucagon, and corticosterone 1 hour after CNO injection. Mice per group n=5-12.

(C) Insulin tolerance after hM3Dq-induced activation of GLP1R or GPR65 vagal afferents. Mice per group n=9-20. Values are presented as mean +/- SEM.

(D) Analysis of plasma corticosterone levels and hepatic *G6pc* mRNA levels from clamp studies. Mice per group n=5-9.

In all experiments, triple transgenic mice and littermate controls were injected with CNO. Mice are from multiple litters. Statistical significance was assessed by two-tailed paired Student's t-test **(A)**, ordinary one-way ANOVA **(B,D)**, or two-way mixed effects ANOVA with Dunnett's test for multiple comparisons **(C)**.

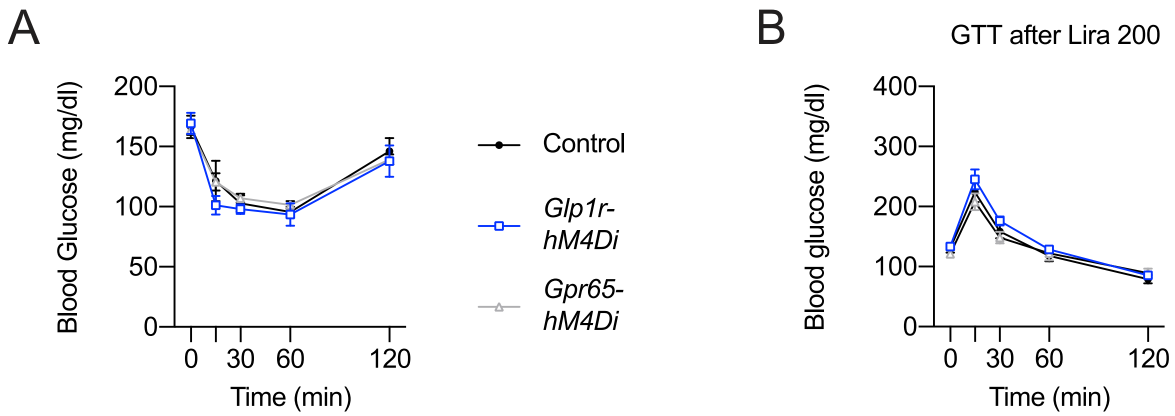

Figure S6, Related to Figure 6: Effects of selective chemogenetic inhibition of GLP1R or GPR65 vagal afferents on glucose metabolism.

(A) Insulin tolerance during hM4Di-induced inhibition of GLP1R or GPR65 vagal afferents. Mice per group n=5-8.

(B) Effects of hM4Di-induced inhibition of GLP1R or GPR65 vagal afferents on glucose tolerance during GTTs. Liraglutide (200 mg/kg) was administered 15 minutes before glucose injections. Mice per group n=4-11.

In all experiments, triple transgenic mice and littermate controls were injected with CNO. Mice are from multiple litters. Statistical significance was assessed by two-way mixed effects ANOVA with Dunnett's test for multiple comparisons. Values are presented as mean +/- SEM.

A

Nav1.8-p2a-Dre::ZsGreen

	ZsGreen / *Nav1.8+*	*Nav1.8+* / ZsGreen
NG	98.33 ± 1.67 %	92.33 ± 7.67 %
DRG	100.00 %	100.00 %

B

Phox2b-Cre::Nav1.8-p2a-Dre::tdTomato and *Wnt1-Cre::Nav1.8-p2a-Dre::tdTomato*

	tdTomato / *Phox2b+*	*Phox2b+* / tdTomato	tdTomato / *Prdm12+*	*Prdm12+* / tdTomato
NG	87.33 ± 4.63 %	100.00 %	-	-
DRG	-	-	98.00 ± 1.16 %	97.67 ± 1.45 %
JG	-	-	91.33 ± 0.88 %	94.33 ± 0.88 %

Glp1r-ires-Cre::Nav1.8-p2a-Dre::tdTomato and *Gpr65-ires-Cre::Nav1.8-p2a-Dre::tdTomato*

	tdTomato / *Glp1r+*	*Glp1r+* / tdTomato	tdTomato / *Gpr65+*	*Gpr65+* / tdTomato
NG	70.33 ± 4.37 %	67.33 ± 9.68 %	96.00 ± 4.00 %	100.00 %

C

Trpv1-ires-Cre::Nav1.8-p2a-Dre::tdTomato, *Sst-ires-Cre::Nav1.8-p2a-Dre::tdTomato*, *Tac1-ires-Cre::Nav1.8-p2a-Dre::tdTomato*, *Vglut3-ires-Cre::Nav1.8-p2a-Dre::tdTomato*

	tdTomato / *Trpv1+*	*Trpv1+* / tdTomato	tdTomato / *Sst+*	*Sst+* / tdTomato	tdTomato / *Tac1+*	*Tac1+* / tdTomato	tdTomato / *Vglut3+*	*Vglut3+* / tdTomato
NG/ JG	84.00 ± 5.77 %	93.67 ± 5.36 %	81.00 ± 19.00 %	78.33 ± 11.67 %	85.00 ± 8.66 %	82.00 ± 6.93 %	-	-
DRG	100.00 %	79.33 ± 7.62 %	67.67 ± 16.90 %	100.00 %	88.67 ± 1.86 %	95.33 ± 0.88 %	60.33 ± 13.86 %	100.00 %

Table S1, related to Figure 1 and S1: Validation of double and triple transgenic mice.

(A-C) Analysis of Zsgreen **(A)**, tdTomato **(B, C)**, and marker gene expression in NG, DRG, and JG. Values are presented as mean +/- SEM.
